# Oseltamivir Phosphate Modulates CD24‐Siglec‐G/10 Interaction to Suppress Microglial‐Driven Neuroinflammation After Cardiac Arrest

**DOI:** 10.1111/cns.70495

**Published:** 2025-08-21

**Authors:** Yushu Chen, Ying Liu, Na Li, Ling Wang, Peijuan Li, Zhangping Sun, Dongping Yu, Ziren Tang, Ping Gong

**Affiliations:** ^1^ Department of Emergency Medicine Shenzhen People's Hospital (The Second Clinical Medical College, Jinan University; the First Affiliated Hospital, School of Medicine, Southern University of Science and Technology) Shenzhen Guangdong China; ^2^ Department of Emergency Medicine First Affiliated Hospital of Dalian Medical University Dalian Liaoning China; ^3^ Department of General Medicine, Xinqiao Hospital Army Medical University Chongqing China; ^4^ Department of Critical Care Medicine Affiliated Hospital of Zunyi Medical University Zunyi Guizhou China; ^5^ Department of Gastrointestinal Surgery Shandong Provincial Hospital Affiliated to Shandong First Medical University Jinan Shandong China; ^6^ Department of Medical Intensive Care Unit (MICU) Zhongshan Hospital Affiliated to Dalian University Dalian Liaoning China; ^7^ Department of Emergency Medicine Second Affiliated Hospital of Dalian Medical University Dalian Liaoning China; ^8^ Department of Emergency Medicine Beijing Chao‐Yang Hospital Affiliated to Capital Medical University Beijing Liaoning China

**Keywords:** cardiac arrest, CD24, microglia, neuroinflammation, NF‐κB, oseltamivir, SiglecG/10, therapeutic targets

## Abstract

**Background:**

In cardiac arrest (CA) patients undergoing cardiopulmonary resuscitation (CPR), neuroinflammation following return of spontaneous circulation (ROSC) contributes to brain ischemia/reperfusion injury and neurological dysfunction. Recent evidence suggested that neuraminidase could exacerbate inflammatory responses by disrupting CD24‐Siglec‐G/10 immune checkpoint axis. As a neuraminidase inhibitor, oseltamivir phosphate (OP) holds potential for immunomodulation beyond its antiviral use. We aimed to investigate the impact and mechanism of OP on neuroinflammation regulation after ROSC.

**Methods:**

Male pigs were randomized into the sham control group, CPR, and CPR + OP group. CA was induced in pigs through 8 min of untreated ventricular fibrillation. Brains were harvested for assessing serum inflammatory markers and neuronal damage at 24 h after ROSC. BV2 microglial underwent oxygen–glucose deprivation/reperfusion (OGD/R). Effects of OP on inflammatory responses, NF‐κB activation, cell viability, and the CD24‐Siglec‐G/10 interaction were evaluated using immunofluorescence, immunoprecipitation, molecular, and biochemical assays.

**Results:**

In vivo, OP attenuated pig cerebral microglial activation and neuronal integrity with attenuated neuroinflammation, alongside time‐dependent neuraminidase activity increases. In vitro, OP suppressed OGD/R‐induced microglial NF‐κB activation, reduced pro‐inflammatory cytokine levels, and preserved CD24‐Siglec‐G interaction, correlating with diminished neuraminidase release.

**Conclusions:**

OP as a repurposed immunomodulator that suppresses microglial‐driven neuroinflammation after CA by preserving sialylation‐dependent CD24‐Siglec‐G/10 interaction.

## Graphical abstract

The schematic diagram demonstrates how oseltamivir phosphate (OP, Tamiflu) alleviates cerebral injury and neuroinflammation after cardiac arrest/cardiopulmonary resuscitation (CA/CPR) through the CD24‐Siglec‐G/10 axis. The interaction between CD24 and Siglec‐G/10 via sialic acid restrains NF‐κB p65 nuclear translocation. This molecular inhibitory mechanism is compromised by neuraminidase during ischemia‐reperfusion injury, while OP exerts neuroprotection through targeted inhibition of desialylation, offering therapeutic opportunities.

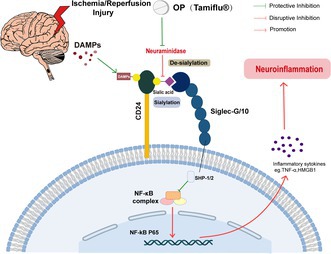

## 1 Introduction

Cardiac arrest (CA) is a major cause of mortality and disability globally [1]. The survival rate for CA patients at the hospital discharge was 10%–30% in Europe and the United States, but only approximately 1.15% in China [2]. Even if patients survived with return of spontaneous circulation (ROSC) after cardiopulmonary resuscitation (CPR), some of them could still develop postcardiac arrest syndrome with lifelong disability [3]. Brain ischemia/reperfusion (I/R) injury, with subsequent neuroinflammation, can significantly impair neurological function and is a key contributor of post‐CA syndrome and death [4, 5]. While the inhibition of inflammation can provide neuroprotection, no targeted drug therapy has proven effective against cerebral I/R damage after ROSC [6]. High mobility group protein B1 (HMGB1), as a damage‐associated molecular pattern (DAMP) molecule, can trigger inflammation through receptors like NOD‐like receptors (NLRs) and Toll‐like receptors (TLR) [7, 8]. This process amplifies the inflammatory response and activates microglia, contributing to the progression of neuroinflammatory conditions [9]. Nuclear factor‐κB (NF‐κB) is a key transcription factor in regulating inflammatory responses and plays a critical role in the process of neuroinflammation induced by HMGB1 activation [10]. Recent studies have highlighted that the interaction between cluster of differentiation 24 (CD24) and Siglec‐G/10 (Siglec‐G in mice and Siglec‐10 in humans and pigs) could negatively regulate this inflammatory response, offering a promising avenue for mitigating inflammation [11, 12, 13, 14].

CD24 is a sialylated glycoprotein and glycophosphatidylinositol‐anchored cell surface receptor expressed across various cell types and tissues, including microglia [15, 16]. CD24 functions as a “don't eat me” signal, inhibiting inflammation and preventing phagocytosis of cancer cells by interacting with Siglec‐G/10 on macrophages [17, 18]. Additionally, CD24 can directly bind to HMGB1, facilitating the formation of a trimeric complex with Siglec‐G/10, which effectively represses inflammatory responses to DAMPs [12, 19]. Siglecs, through sialic acid‐based pattern recognition, can negatively regulate both adaptive and innate immune reactions to inhibit immune pathways by recognizing self‐glycan ligands and recruiting SHP‐1 and SHP‐2 phosphatases [20, 21]. By selectively modifying innate immune DAMP reactions rather than pathogen‐associated molecular patterns (PAMPs), Siglec‐G/10 and CD24 could facilitate the differentiation between infectious agents and tissue damage by the innate immune system [11, 22, 23]. Furthermore, the binding of CD24 to Siglec‐G/10 could mitigate DAMP‐related inflammation by suppressing the triggering of HMGB1/TLR4/NF‐κB pathway [7, 23].

Neuraminidases act as virulence factors by desialylating CD24, which reduces its binding to Siglec‐G and enhances immune reactions, exacerbating HMGB1‐induced inflammation [24]. By preventing the desialylation of CD24, neuraminidase inhibitor preserves its interaction with Siglec‐G/10, thereby dampening excessive inflammation [21, 25, 26]. This mechanism is particularly relevant in sepsis, where HMGB1‐induced inflammation is a key pathogenic factor [12]. Therefore, neuraminidase inhibitor may regulate the immune responses and prevent the CD24‐Siglec‐G/10 pathway activation.

Oseltamivir phosphate (OP), a widely used neuraminidase inhibitor, can not only limit viral spread but also modulate immune responses [27, 28, 29]. However, whether OP could reduce neural damage and neuroinflammation after ROSC, as well as its underlying pathophysiological impact, was not reported previously. In the current study, we investigated the effects of OP on the neuraminidase‐modulated disruption of the CD24‐Siglec‐G/10 interaction to mitigate neuroinflammation after ROSC. The study results could pave the way for novel therapeutic strategies to improve outcomes following post‐CA brain injury.

## 2 Materials and Methods

### 2.1 Animal

The experimental protocol was approved by the Ethics Committee of Dalian Medical University (#AEE19096). Fifteen male Bama minipigs (*Sus scrofa domestica*), aged 4–6 months and weighing 30.1 ± 2.0 kg, were obtained from the university Animal Experiment Center.

### 2.2 CA/CPR Model

The CA/CPR animal model was established based on a previously published method, with minor modifications [30, 31, 32]. Pigs were fasted overnight and, on the day of the experiment, received intramuscular injections of midazolam (0.4 mg/kg) and butorphanol (0.2 mg/kg, Jiangsu Nhwa Pharmaceutical, Jiangsu, China) for anesthesia. After 5–10 min, 2 mg/kg propofol was given intravenously through a vein in the ear. The pigs were in a supine position on a V‐shaped surgical table. A standard lead II electrocardiogram (ECG) was obtained after attaching electrodes to the shaved skin of both upper limbs and the left lower limb.

Following complete anesthesia, tracheal intubation was performed. Mechanical ventilation under synchronized intermittent mandatory mode was initiated on a VELA ventilator (CareFusion, CA, USA), with the settings of respiration rate 12–20 breaths/min, tidal volume 6–7 mL/kg, pressure support 10 cmH_2_O, and fraction of inspired oxygen 21%. The end‐tidal carbon dioxide partial pressure was maintained at 35–45 mmHg and monitored by a capnometer (Respironics, PA, USA). Anesthesia was continued by intravenous infusions of fentanyl (5 mg/kg/h, Jiangsu Nhwa Pharmaceutical, Jiangsu, China) and pentobarbital (8 mg/kg/h, Sigma‐Aldrich, St. Louis, MO, USA), with additional doses of propofol (0.4 mg/kg) given when required.

After skin disinfection, a pulse index continuous cardiac output (PiCCO) catheter (Pulsion Medical Systems, Munich, Germany) equipped with a solid‐state thermistor tip and temperature sensor (PULSION Medical Systems SE, Munich, Germany) was applied to cannulate the right femoral artery. The cardiac index and arterial pressure were monitored. A 7F triple‐lumen central venous catheter (Royal Fornia Medical Equipment, Zhuhai, China) was inserted into the right femoral vein for medication administration, fluid infusion, and venous blood sampling. Right atrial pressure was monitored by a 7F three‐lumen central venous catheter placed in the left internal jugular vein. Ventricular fibrillation (VF) was triggered by a 5‐Fr pacing catheter (Boston Scientific, MA, USA) advanced into the right ventricle through the right internal jugular vein. All procedures were performed under aseptic conditions. Heparinized normal saline (5 U/mL) was applied to prewash all catheters to prevent clotting. Body temperature was monitored by a temperature‐sensing indwelling Foley catheter (Integral Medical Products, Shaoxing, China) placed in the bladder. Cardiac rhythm and hemodynamics were continuously monitored by a IntelliVue MP20 monitor (Philips, Boeblingen, Germany).

After catheterization, baseline measurements were documented after the animals were stabilized for 30 min. VF was induced by applying a progressively increasing 60‐Hz current at a maximum 2.5 mA electric shock to the right ventricle. Mechanical ventilation was stopped once the VF was confirmed by the appearance of VF waves on the ECG and a sharp drop in blood pressure. Eight minutes after leaving VF untreated, CPR was initiated with a compression‐to‐ventilation ratio of 30:2. Ventilations were performed through the endotracheal tube by a bag respirator with room air. After 2 min of CPR, a biphasic electrical shock (2–4 J/kg) was delivered using a biphasic defibrillator (Philips, MA, USA). If no ROSC occurred, an additional 2 min CPR was given, which was followed by intravenous administration of epinephrine (30 mg/kg) every 3 min until ROSC. ROSC was defined as rhythmic heartbeats sustained for ≥ 20 min, with a mean arterial pressure > 60 mmHg. Mechanical ventilation was resumed the same as the baseline settings once ROSC was achieved. Otherwise, CPR was discontinued if there was no ROSC after resuscitation for 20 min.

### 2.3 Experimental Protocol

Pigs were randomly assigned into three groups using a sealed envelope method (*n* = 5 per group): a sham group, without CA/CPR and OP administration; a CPR group, with CA/CPR and no OP administration; and an OP group, with 5 mg/kg OP (Tamiflu; Roche, Switzerland) administered immediately via nasogastric feeding tube within 2 min after ROSC was achieved and another dose given 12 h later. The sample size was determined based on Neurological Deficit Scale (NDS) data from a prior porcine CA/CPR study [33], targeting 90% power (α = 0.05, effect size = 1.5). The calculation yielded a minimum requirement of *n* = 4 per group. Considering potential attrition (e.g., failed ROSC), we included *n* = 5 per group.

Baseline physiological parameters, including body weight, bladder temperature, heart rate, mean aortic pressure, arterial lactate, end‐tidal carbon dioxide, and arterial oxygen partial pressure, were collected. Arterial blood was sampled at several time points, including baseline (pre‐CA induction), 30 min, 6 h, 12 h, and 24 h post‐ROSC. After centrifuging for 15 min at 1000 ***g*** and 4°C, serum supernatant aliquots were obtained for analysis. At 24 h post‐ROSC, neurological function was evaluated using NDS [31]. The NDS scale consisted of five parameters: respiratory patterns, behavioral responses, motor functions, sensory functions, and consciousness levels. The total score was obtained by adding the scores of the individual components. A score of zero implied normal neurological function, and a score of 400 suggested brain death. Investigators remained blinded to therapeutic interventions. Animals were then euthanized by intravenous injection of propofol (3 mg/kg) and potassium chloride (10–20 mL, 10 mol/L). The left hemisphere frontal cortex, due to its critical role in functional outcomes, was dissected, fresh‐frozen in liquid nitrogen, and stored at −80°C for further analysis.

### 2.4 Oxygen–Glucose Deprivation/Reperfusion (OGD/R) Model and Transfection

Murine BV2 microglia were cultured in Dulbecco's modified Eagle's medium (DMEM) (Thermo Fisher Scientific, MA, USA) with 100 μg/mL penicillin/streptomycin and 10% heat‐inactivated fetal bovine serum (Changsha Serbox Biotechnology, China). Cells were incubated in a humidified environment with 5% CO_2_ at 37°C.

An in vitro OGD/R model mimicking CA/CPR was established as previously described [33]. Briefly, after rinsed with phosphate‐buffered saline (PBS) three times, cells were incubated in prewarmed for 10 min in glucose‐free DMEM (Thermo Fisher Scientific, MA, USA). OGD was induced by transferring cells into an anaerobic chamber (Billups Rothenberg, CA, USA) containing 95% N_2_ and 5% CO_2_ at 37°C for 2 h. After OGD, normal glucose‐containing DMEM was used to replace the glucose‐free DMEM to let cells return to normoxic conditions (5% CO_2_, 95% air) for 22 h of reperfusion.

CD24 and Siglec‐G small‐interfering RNA (siRNA) or pcDNA 3.1(+) overexpression plasmids were synthesized by Suzhou Jima Gene Co. Ltd. (China). The transfect sequences are listed in Table S1. BV2 cells were divided into five experimental groups, including (1) Normal control (NC): cells cultured for 24 h under standard conditions; (2) OGD/R group: cells subjected to OGD/R as described above; (3) si‐*CD24‐Siglec‐G* group: cells transfected with CD24/Siglec‐G siRNA using Lipofectamine 3000 (L3000015, Thermo Fisher Scientific) on day 2 of culture, followed by OGD/R; (4) pcDNA3.1(+)‐*CD24‐Siglec‐G* groups: cells transfected with CD24/Siglec‐G overexpression plasmids using Lipofectamine 3000, followed by OGD/R; (5) OGD/*R* + OP group: cells treated with 40 μM OP (MCE, Monmouth Junction, NJ, USA) after OGD/R.

### 2.5 Quantitative Real‐Time Polymerase Chain Reaction (RT‐qPCR)

A commercial total RNA extraction kit (9767, Takara, Beijing, China) was used to extract total RNA from brain tissues. RNA purity and concentration were determined using a NanoDrop spectrophotometer (Thermo Fisher Scientific, MA, USA).

The Prime Script RT reagent Kit with gDNA Eraser (RR047Q, TaKaRa, Beijing, China) was used to reverse‐transcribe the total RNA (10 ng) into cDNA and eliminate genomic DNA contamination. RT‐qPCR reactions were performed in 20 μL volumes with TB Green Premix Ex Taq II (RR820Q, TaKaRa, Beijing, China) on an RT‐PCR system (Step One, Thermo Fisher Scientific, MA, USA). Thermocycling was set as initial denaturation at 95°C for 30 s, amplification for 40 cycles at 95°C for 5 s and 60°C for 34 s, and melting curve at 95°C for 15 s, 60°C for 1 min, and then a slow raising of temperature to 95°C. The 2^−ΔΔCt^ method was applied to calculate the relative gene expression [34], with β‐actin set as the endogenous control. The sequences for the primer sequences are listed in Table S2. All experiments included three technical replicates.

### 2.6 Cell Viability Assay

The Cell Counting Kit‐8 (CCK‐8) (APExBIO, TX, USA) was used to determine the cell viability. Briefly, BV2 cells were seeded in96‐well plates at a density of 10,000/well and cultured overnight. On day 2, cells were subjected to OGD/R by incubation in serum‐ and glucose‐free DMEM under hypoxic conditions (5% CO_2_, 95% N_2_) at 37°C for 2 h. After OGD/R, cells were treated with OP (0–180 μM) for 24 h or 48 h under normoxic reperfusion conditions. Dose optimization was performed using 0–100 μM OP (Figure S1). Next, each well with 100 μL of fresh DMEM was added 10 μL of CCK‐8 solution. Cells were then incubated for 1.5 h at 37°C. A microplate reader (Molecular Devices, CA, USA) was used to record optical density (OD) at 450 nm. Experiments were repeated in duplicate. Cell viability was calculated using the following formula: Cell viability (%) = (OD_treatment group_ – OD_blank group_)/(OD_control group_ – OD_blank group_) × 100%.

### 2.7 Neuraminidase Activity Assay

The Amplex Red Neuraminidase Assay Kit (A22178, Thermo Fisher, USA) was applied to measure the neuraminidase activity. The assay quantifies hydrogen peroxide (H_2_O_2_) generated through the enzymatic reaction: neuraminidase hydrolyzes sialic acid residues from glycoconjugates to release desialylated galactose, which is subsequently oxidized by galactose oxidase to produce H_2_O_2_. In the presence of HRP, H_2_O_2_ reacts with Amplex Red to generate the fluorescent product resorufin (excitation/emission: 571/585 nm). Samples were incubated with the reaction mixture for 30 min at 37°C, and then subjected to a Synergy X Multi‐Mode Microplate Reader (TECAN Infinite 200 PRO, Männedorf, Switzerland) to determine the fluorescence intensity.

### 2.8 Enzyme‐Linked Immunosorbent Assay (ELISA)

Supernatants from BV_2_ cells and serum samples were collected. Pig serum HMGB1, TNF‐α, and NSE levels were determined by commercial ELISA kits (HMGB1: #EP0301; TNF‐α: #EP0159; NSE: #EP0387; Fine Test, Wuhan, China), and BV2 cell supernatant HMGB1, IL‐6, and TNF‐α levels were quantified by commercial ELISA kits (HMGB1: SYP‐M0467; IL‐6: SYP‐M0031; TNF‐α: SYP‐M0036; UpingBio, Wuhan, China).

### 2.9 Co‐Immunoprecipitation and Western Blot

For co‐immunoprecipitation assays, whole cell lysates were prepared using NP‐40 lysis (HY‐Y1884, MCE) buffer containing 1 × protease inhibitor cocktail (PR20016, Proteintech, IL, USA). Lysates were incubated on a rotary shaker for 30 min at 4°C. After centrifugation for 20 min at 12,000 ***g*** and 4°C, supernatant was incubated with primary antibodies or IgG control on a rocker at 4°C overnight. Then, the supernatant was incubated with protein A/G agarose beads (HY‐K0202, MCE) for 4 h at 4°C. After washing the beads bound to antigen–antibody complexes with phosphate buffered saline containing 0.1% Tween 20 (PBST) three times, immunoprecipitated proteins were separated in SDS‐PAGE and subjected for Western blot analysis.

In the Western blot analysis, RIPA buffer with a 100× protease inhibitor cocktail was used to lyse brain tissues and BV2 cells. Cytoplasmic and nuclear proteins were obtained using a commercial extraction kit (P0027, Beyotime, China) and their concentrations were calculated via the bicinchoninic acid assay (P0012, Beyotime, China). Equal protein amounts (30 μg/lane) were loaded and separated on 10% SDS‐PAGE gels and transferred onto polyvinylidene fluoride membranes (Millipore Sigma, MA, USA). After standard blocking and primary and secondary antibody incubations, chemiluminescent signals were developed using an HRP substrate kit (WP20005, Thermo Fisher, USA) and captured with a gel imaging system. Band intensity was quantified using ImageJ software (NIH, MD, USA). The primary antibodies used in this study are provided in Table S3.

### 2.10 Hematoxylin–Eosin (H&E) Staining

The collected pig brain tissues were fixed with 4% paraformaldehyde (PFA) and embedded in paraffin for each section. After dewaxing, the sections (5 μm) were first immersed in 95% and 70% ethanol for 1 min each and rinsed by double‐distilled water (ddH_2_O). Nuclear staining was performed with hematoxylin (H8070; Solarbio) for 2 min. Then, it was differentiated in 0.3% acid alcohol. After three cycles of ddH_2_O rinsing, cytoplasmic counterstaining was submerged and stained in eosin staining solution (G1100; Solarbio) for 2 min. Finally, after dehydrating and sealing, the section staining was observed and photographed under a microscope (Olympus BX43, Tokyo, Japan).

### 2.11 Immunofluorescence Staining Analysis

At room temperature, brain tissue sections (5 μm) or BV2 cells were washed three times with PBS, fixed for 15 min in 4% PFA, and blocked for 30 min with 5% goat serum (C0265, Beyotime, Shanghai, China). For dual‐labeling experiments, sections were incubated at 4°C overnight with the rabbit anti‐Iba1 (1:200; 10,904‐1‐AP; Proteintech) co‐stained with mouse anti‐HMGB1 (1:800; ab190377; Abcam) primary antibodies. Both BV2 cells and tissue sections were subjected to rabbit anti‐p65 antibody (1:500; #6956; CST) incubation. Then, these samples were treated with secondary antibodies (goat anti‐rabbit or goat anti‐mouse) conjugated to Alexa Fluor 488 or Alexa Fluor 594 (A‐11001; Thermo Fisher, USA). The 4′,6‐diamidino‐2‐phenylindole (DAPI) was used to stain the cell nuclei for observation by a fluorescence microscope.

### 2.12 Nissl Staining

Nissl staining was performed using a commercial kit (G1086; Servicebio, Wuhan, China). Briefly, tissue sections (5 μm) were soaked in Nissl staining solution A for 15 min and quickly rinsed with ddH_2_O. Sections were then differentiated in Nissl staining solution B and observed under a light microscope until Nissl bodies were distinctly visible and background staining was minimized. Next, gradient ethanol was used to dehydrate the sections, which were cleared in xylene for 2 min. Five random fields per section were quantified. The mean number of Nissl bodies was analyzed using ImageJ software.

### 2.13 Cell Surface α2,6‐Linked Sialic Acid Fluorescence of Different Concentration OP Analyzed by SNA‐FITC

During OGD/R, α2,6‐linked sialic acid residues were removed from the BV2 cell surface after 12.5 U/mL neuraminidase (HY‐P2988, MCE) treatment, as previously described [35]. Cells were assigned into eight experimental groups: unstained control, untreated control, OGD/R, OGD/*R*+ neuraminidase, and OGD/*R*+ 40, 60, 80, and 100 μmol/L OP. For fluorescence staining, cells were fixed with 4% PFA for 15 min at room temperature, and blocked with 10% bovine serum albumin (BSA) for 30 min. Next, *Sambucus nigra* Lectin (SNA)‐FITC (L32479, Thermo‐Fisher, USA) was diluted 1:300 in 5% BSA and incubated overnight at 4°C. Each step was followed with PBS washes (5 min each) three times. Finally, DAPI was used to stain the cell nuclei for observation by a fluorescence microscope.

For flow cytometry analysis, BV2 cells were subjected to trypsin digestion, centrifugation for 5 min at 300 ***g***, and twice PBS washes. Cells were resuspended in 50 μL PBS (unstained group) and 49.5 μL PBS+0.5 μL SNA‐FITC (remaining seven groups). After incubation for 30 min at 4°C in the dark, PBS wash, and centrifugation, cell surface fluorescence intensity was measured in flow cytometry with modifications from a published protocol [36].

### 2.14 Statistical Analysis

All statistical analyses were performed in GraphPad Prism 8 (GraphPad Software, CA, USA) and SPSS v22.0 (IBM, NY, USA). Data were presented as means ± standard error of the mean (SEM). All quantitative data were assessed for normality distribution using Shapiro–Wilk tests. Homogeneity of variance was verified using Levene's test. For data satisfying both normality and variance homogeneity assumptions, parametric tests were employed. Otherwise, a nonparametric test was applied. One‐way ANOVA was used for comparisons among groups, whereas two‐way ANOVA assessed the effects of group and time. Significant outcomes were further analyzed with Bonferroni‐corrected post hoc tests. Statistical significance was set at a *p* < 0.05.

## 3 Results

### 3.1 OP Alleviates Neuronal Damage and Neuroinflammation Post‐ROSC

**FIGURE 1:**
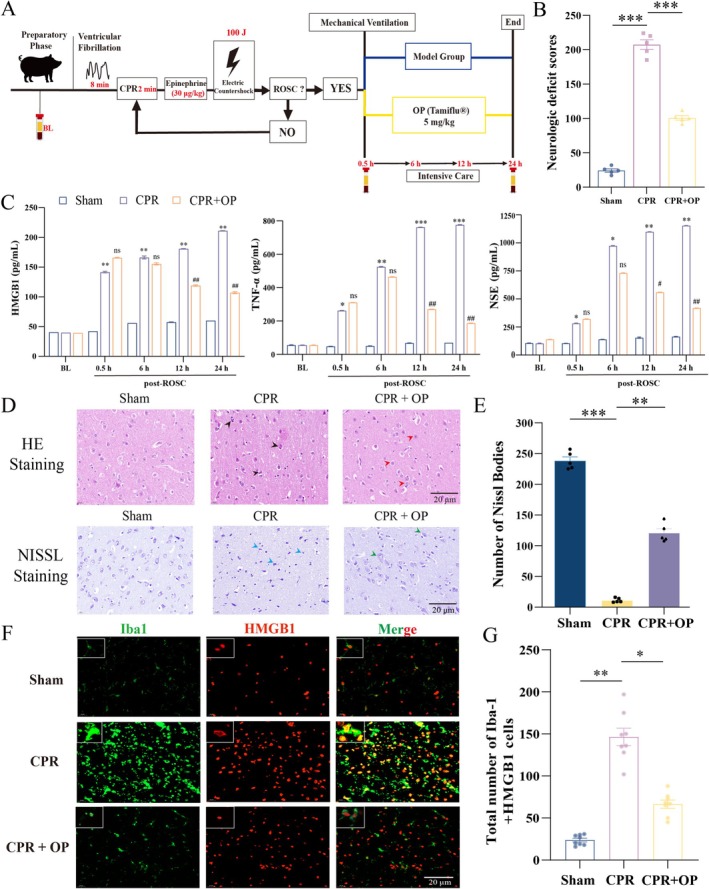
OP alleviates neuronal damage and neuroinflammation after ROSC. (A) The experimental flow diagram for a pig model creation with cardiac arrest/cardiopulmonary resuscitation (CA/CPR) and postresuscitation interventions. (B) The neurologic deficit score (NDS) was assessed at 24 h post‐ROSC. (C) Serum levels of high‐mobility group box 1 (HMGB1), tumor necrosis factor‐α (TNF‐α), and neuron‐specific enolase (NSE) quantified by ELISA in Sham, CPR, and CPR + OP groups at post‐ROSC 0.5, 6, 12, and 24 h. *p < 0.05, **p < 0.01, ***p < 0.001 vs. BL in CPR group. ^#^ *p* < 0.05, ^##^ *p* < 0.01 vs. same time point in CPR group. (D) Representative histological images of Hematoxylin and Eosin (H&E) and Nissl staining in cortical brain tissues from Sham, CPR, and CPR + OP groups. Neuronal shrinkage (black arrows) and neuronal structural integrity (red arrows) are indicated in H&E staining. Damaged or distorted neurons (blue arrows) and preserved Nissl bodies (green arrows) are indicated in Nissl staining. Scale bar: 20 μm. (E) Quantitative analysis of Nissl body number in the cerebral cortex (mean ± SEM). (F) Immunofluorescence images showing Iba1 (green) and HMGB1 (red) localization in brain tissue. Scale bar: 20 μm. (G) Quantification of Iba1 and HMGB1 fluorescence intensity (mean ± SEM). **p < 0.01, ***p < 0.001 (one‐way ANOVA with Bonferroni post hoc test).

Pigs subjected to 8‐min CA/CPR modeling were assigned randomly to three groups, including the OP‐treated group (5 mg/kg), sham group, and CPR model group (Figure 1A). No significant differences in the baseline characteristics (body weight, bladder temperature, heart rate, mean aortic pressure, arterial lactate, end‐tidal carbon dioxide, and arterial oxygen partial pressure) were found among the three groups (all *p* > 0.05, Table S4). NDS was significantly lower in the CPR + OP group than that in the CPR group at 24 h post‐ROSC (*p* < 0.001, Figure 1B). ELISA revealed time‐dependent changes in HMGB1, TNF‐α, and NSE levels (Figure 1C). Compared with baseline levels, both the CPR and CPR + OP groups showed significantly elevated serum levels of HMGB1, TNF‐α, and NSE during the first 6 h post‐ROSC (all *p* < 0.01). Within the CPR group, serum HMGB1, TNF‐α, and NSE levels further increased significantly at 12 h and 24 h post‐ROSC compared with 0.5 h (all *p* < 0.01). Moreover, HMGB1, NSE, and TNF‐α levels at 24 h were significantly higher than those at 6 h (*p* < 0.01 for HMGB1 and NSE; *p* < 0.001 for TNF‐α). In contrast, biomarker levels in the CPR + OP group peaked at 6 h post‐ROSC. Subsequently, OP treatment induced a progressive decline in these biomarkers, resulting in significantly lower levels at both 12 h and 24 h compared with the 6 h peak (HMGB1 and TNF‐α *p* < 0.01 at both 12 h and 24 h; NSE *p* < 0.05 at 12 h, *p* < 0.01 at 24 h). H&E staining revealed that CPR group neurons displayed eosinophilic shrinkage, nuclear pyknosis, and disrupted laminar architecture (black arrows, Figure 1D). OP preserved neuronal morphological integrity (red arrows). In Nissl staining at 24 h post‐ROSC, CPR group neurons exhibited shrunken nuclei and condensed Nissl bodies (blue arrows), whereas OP‐treated brains showed intact Nissl bodies (green arrows). Quantitation suggested that OP significantly increased the number of Nissl bodies (*p* < 0.01 vs. CPR group, Figure 1E). As illustrated in the magnification images (upper left panel), immunofluorescence demonstrated nuclear HMGB1 localization in sham microglia (ramified morphology), contrasting with cytoplasmic HMGB1 translocation in activated CPR microglia (Figure 1F). Quantitative analysis confirmed that Iba1^+^/HMGB1^+^ cells colocalize (yellow signal) decreased by 2.23 ± 1.1‐fold after OP treatment (*p* < 0.05 vs. OGD/R; Figure 1G), indicating suppressed microglial activation with subsequent HMGB1 release.

### 3.2 OP Inhibits NF‐κB Pathway Activation and Reduces Pro‐Inflammation Cytokines Expression Post‐ROSC

**FIGURE 2:**
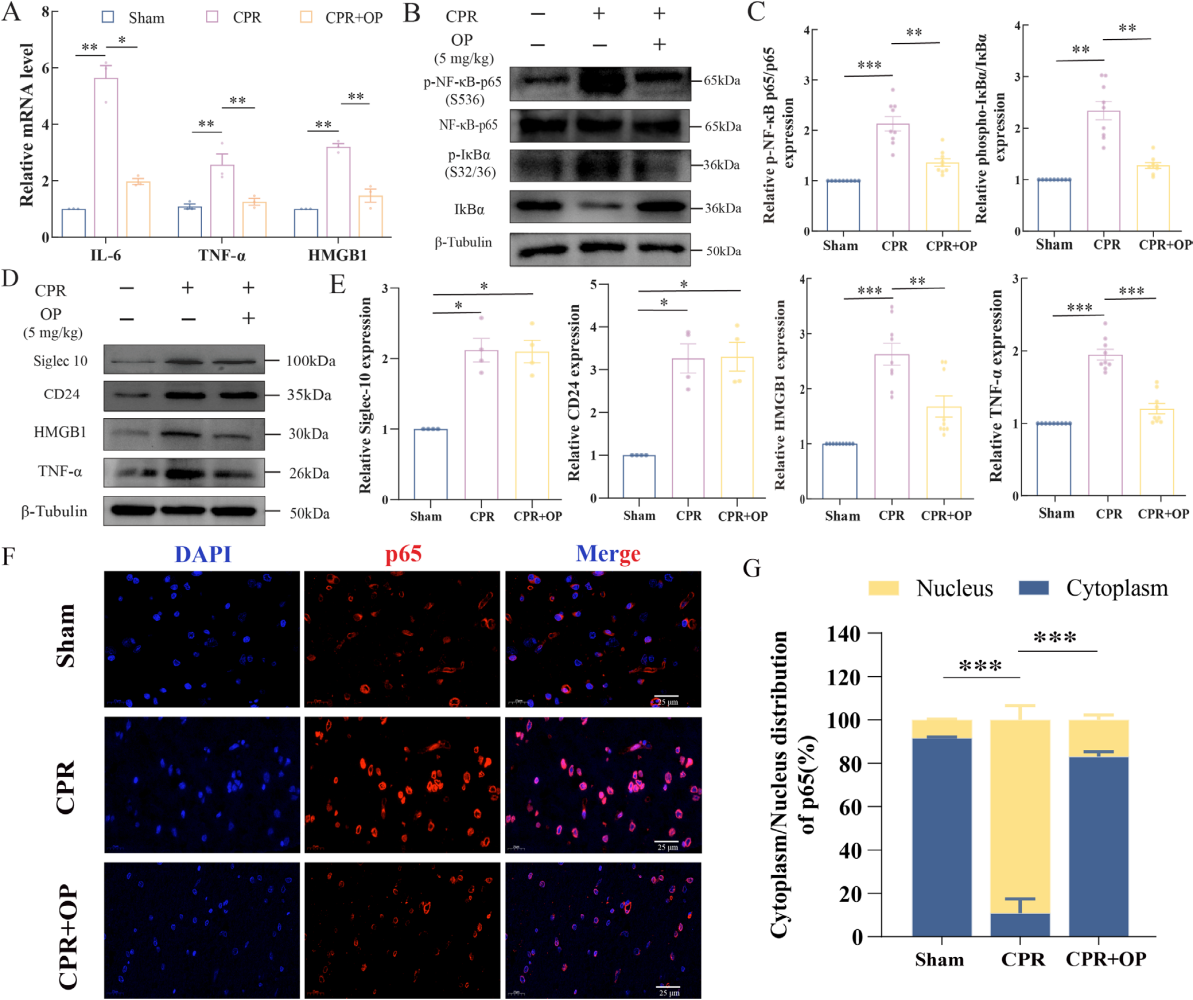
OP suppresses NF‐κB pathway activation and pro‐inflammation cytokines expression in vivo. (A) Relative mRNA levels of interleukin‐6 (IL‐6), TNF‐α, and HMGB1 in CPR and CPR + OP groups, measured by quantitative reverse transcription‐PCR (qRT‐PCR) (normalized to β‐actin, *n* = 3). (B, C) Representative Western blot (B) and quantitative analysis (C) of total NF‐κB p65, phosphorylated NF‐κB p65 (Ser536), total IκBα, and phosphorylated IκBα (Ser32/36) in pig brain tissues from CPR and CPR + OP groups. (D, E) Western blot (D) and quantification (E) of CD24, Siglec‐10, HMGB1, and TNF‐α protein levels in pig brain tissues from CPR and CPR + OP groups. (F) Immunofluorescence images illustrating NF‐κB p65 subcellular localization in the cerebral cortex. Nuclei are counterstained with DAPI (blue), p65 is labeled with an anti‐p65 antibody (red), and merged images depict nuclear translocation. Scale bar: 25 μm. (G) Quantification of cytoplasmic/nuclear p65 ratio in Sham, CPR, and CPR + OP. Data reported as the percentage of nuclear p65 distribution (mean ± SEM). *p < 0.05, **p < 0.01, and ***p < 0.001 (one‐way ANOVA with Tukey's post hoc test).

To explore the potential inflammatory suppressive mechanism of OP, we analyzed NF‐κB signaling and cytokine profiles in post‐ROSC brain tissue. At 24 h post‐ROSC, OP‐treated pigs showed significantly lower TNF‐α, IL‐6, and HMGB1 mRNA levels compared to the CPR pigs (all *p* < 0.01, Figure 2A). Western blot analysis revealed that OP suppressed CA‐induced phosphorylation of IκBα (Ser32/36, p‐IκBα) and NF‐κB p65 (Ser536, p‐p65) (all *p* < 0.01 vs. CPR group; Figure 2B,C). Consistent with mRNA data, OP treatment also reduced HMGB1 (*p* < 0.001) and TNF‐α (*p* < 0.01) protein concentrations (Figure 2D,E). In addition, both CD24 and Siglec‐10 protein levels were upregulated in the CPR group and remained unchanged after OP intervention (all *p* < 0.05 vs. Sham; Figure 2D,E). Immunofluorescence quantification demonstrated a 71.9% ± 2.9% increase in nuclear p65 accumulation in the CPR group versus Sham (*p* < 0.001). OP treatment reduced this accumulation by 62.61% ± 4.32% (*p* < 0.001 vs. CPR group; Figure 2F,G). These findings indicated that OP could inhibit IκBα phosphorylation to block NF‐κB p65 nuclear translocation and suppress TNF‐α and HMGB1 expression, thereby attenuating neuroinflammation.

### 3.3 Neuraminidase Activation

**FIGURE 3:**
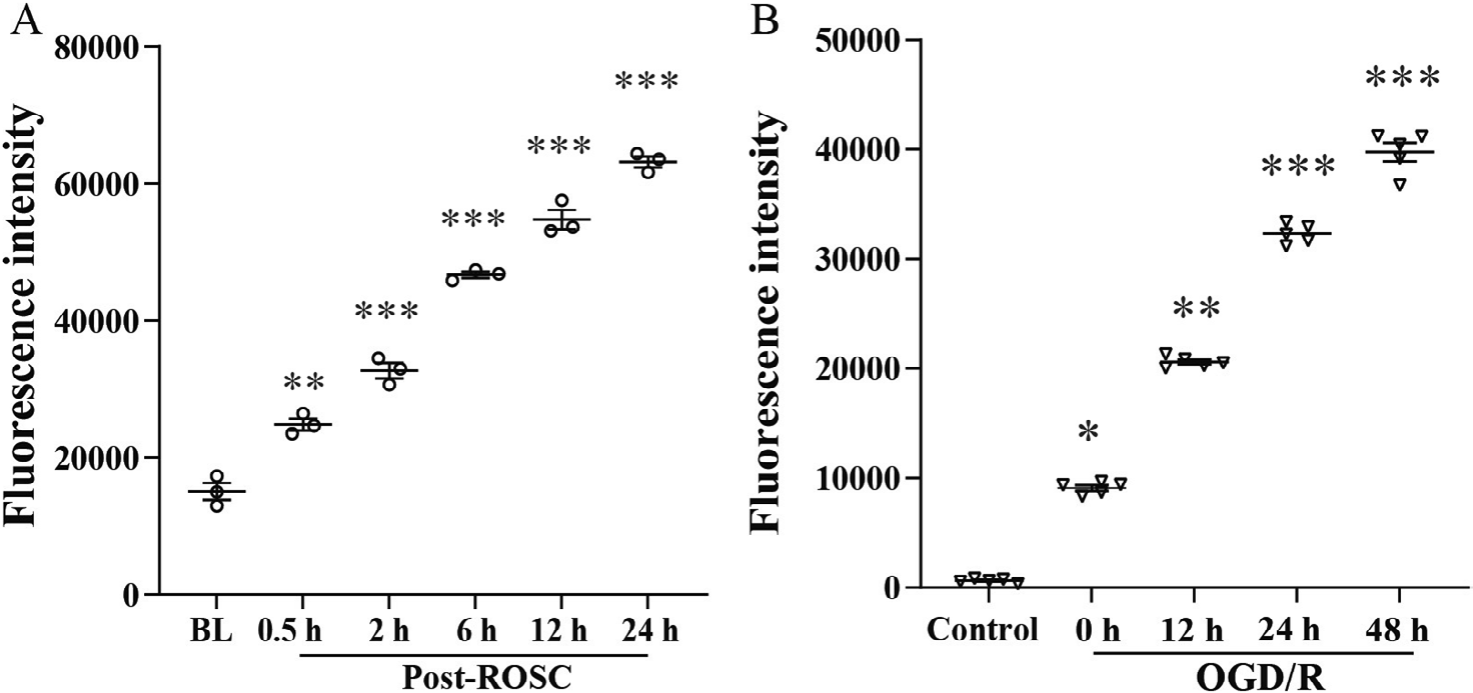
Neuraminidase activity is upregulated in vivo and in vitro following ischemic injury. (A) Neuraminidase activity in pig serum after CA/CPR, measured at post‐ROSC 0.5, 6, 12, and 24 h. (B) Neuraminidase activity in BV2 microglial cell culture supernatant following oxygen–glucose deprivation/reperfusion (OGD/R), assessed at 0, 12, 24, and 48 h postreperfusion. Both models exhibited a time‐dependent upward trend in neuraminidase activity, peaking at post‐ROSC 24 h (serum) and 48 h (cell supernatant). Data are reported as mean ± SEM. *p < 0.05, **p < 0.01, ***p < 0.001 vs. BL/Control group (two‐way ANOVA with Bonferroni post hoc test).

To investigate the role of neuraminidase in OP‐mediated neuroprotection, we quantified neuraminidase activity in both in vivo and in vitro models. In in vivo experiments, serum neuraminidase activity in post‐CA pigs increased time‐dependently within 24 h post‐ROSC, peaking at 4.24 ± 0.71‐fold higher than baseline levels (*p* < 0.001, Figure 3A). In BV2 microglia subjected to OGD/R, neuraminidase levels in cell culture supernatants progressively increased over 48 h, reaching 4.17 ± 0.54‐fold higher than controls (*p* < 0.001; Figure 3B). These results demonstrated that cerebral I/R injury could trigger significant neuraminidase activation in both systemic and cellular contexts, likely through enhanced cleavage of sialic acid residues from glycoconjugates to drive inflammatory cascades.

### 3.4 Effect of OP Treatment on BV2 Microglia Viability and α2,6‐Sialic Acid Expression

**FIGURE 4:**
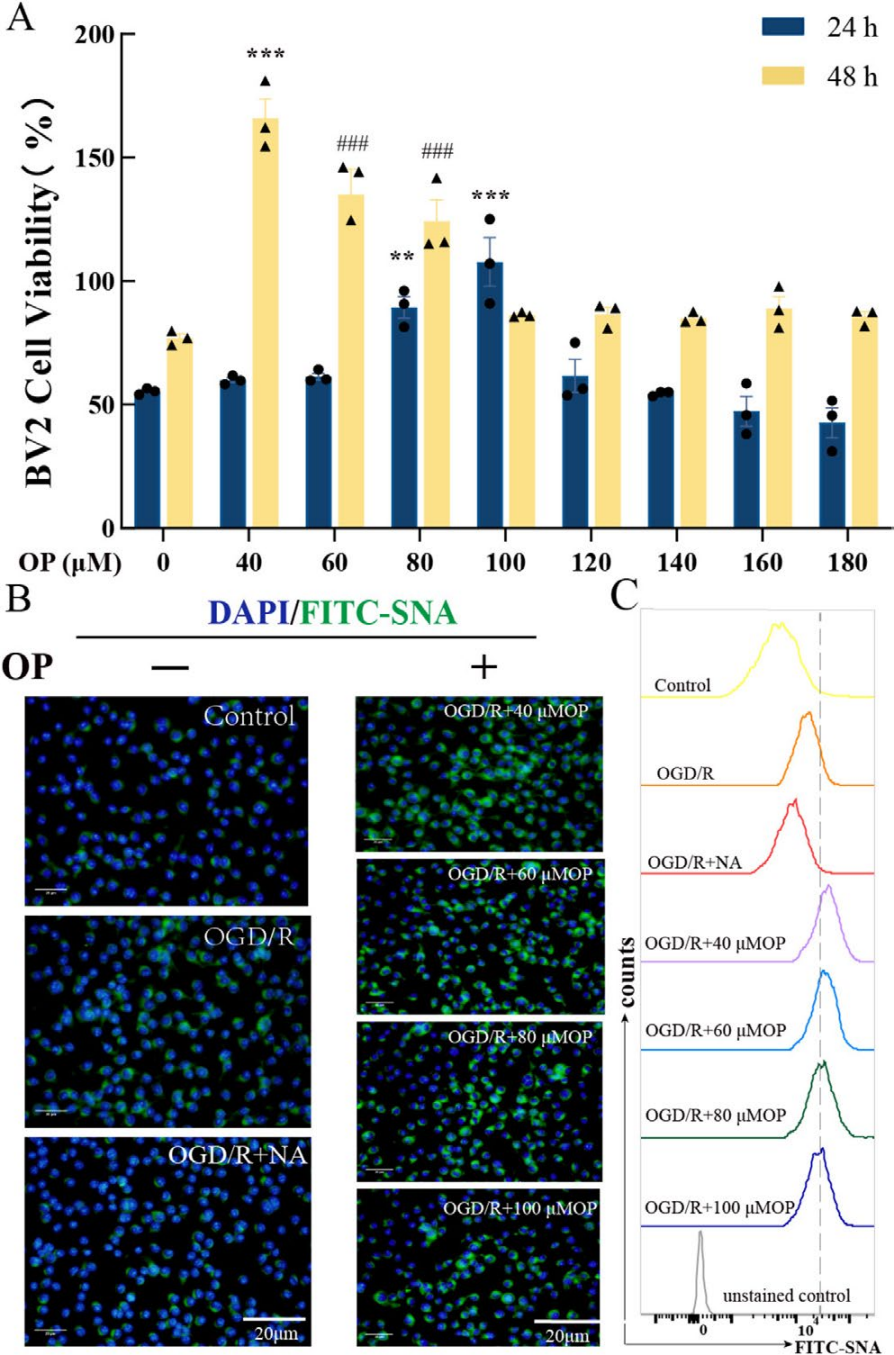
OP enhances microglial viability and preserves α2,6‐sialic acid expression in BV2 cells following OGD/R. (A) Cell viability of BV2 microglia treated with increasing OP concentrations (0–180 μM) at 24 h and 48 h post‐OGD/R, assessed using the Cell Counting Kit‐8 (CCK‐8) assay. Data are presented as mean ± SEM. **p < 0.01, ***p < 0.001 vs. 24/48 h untreated group. ^###^ *p* < 0.001 vs. 40 μM OP to 48 h group (two‐way ANOVA with Bonferroni post hoc test). (B) α2,6‐linked sialic acid expression on BV2 microglia surface, evaluated by SNA‐FITC fluorescence staining (green) and flow cytometry. Representative fluorescent images (40× magnification) show SNA‐FITC signal intensity. Nuclei (blue) counterstained with DAPI. Scale bar: 20 μm. (C) Flow cytometry was used to assess the levels of α2,6‑sialylation in the different treatment groups. NA, neuraminidase; OP, oseltamivir phosphate.

BV2 microglia viability was assessed at 24 h and 48 h post‐OGD/R using CCK‐8 assays in different OP concentrations (0–180 μM), revealing time‐dependent responses (Figure 4A). At 24 h post‐OGD/R, 80 μM and 100 μM OP increased viability by 1.68 ± 0.42‐fold and 1.90 ± 0.36‐fold, respectively (all *p* < 0.001 vs. untreated group). At 48 h post‐OGD/R, treatments of BV2 cells with 40 μM OP significantly enhanced cell viability higher than all other concentrations (2.27‐fold increase, all *p* < 0.001). Based on sustained pro‐survival efficacy at 48 h, 40 μM OP was selected for subsequent experiments.

To evaluate α2,6‐sialylation modulation, *Sambucus nigra* lectin (SNA)‐FITC staining and flow cytometry were employed (Figure 4B,C). Exogenous neuraminidase reduced α2,6‐sialic acid levels relative to the OGD/R group. OP treatment (40–100 μM) dose‐dependently restored sialylation, with 40 μM OP achieving maximal preservation.

### 3.5 Suppression of OP on NA‐Induced Activation in NF‐κB Pathway

**FIGURE 5:**
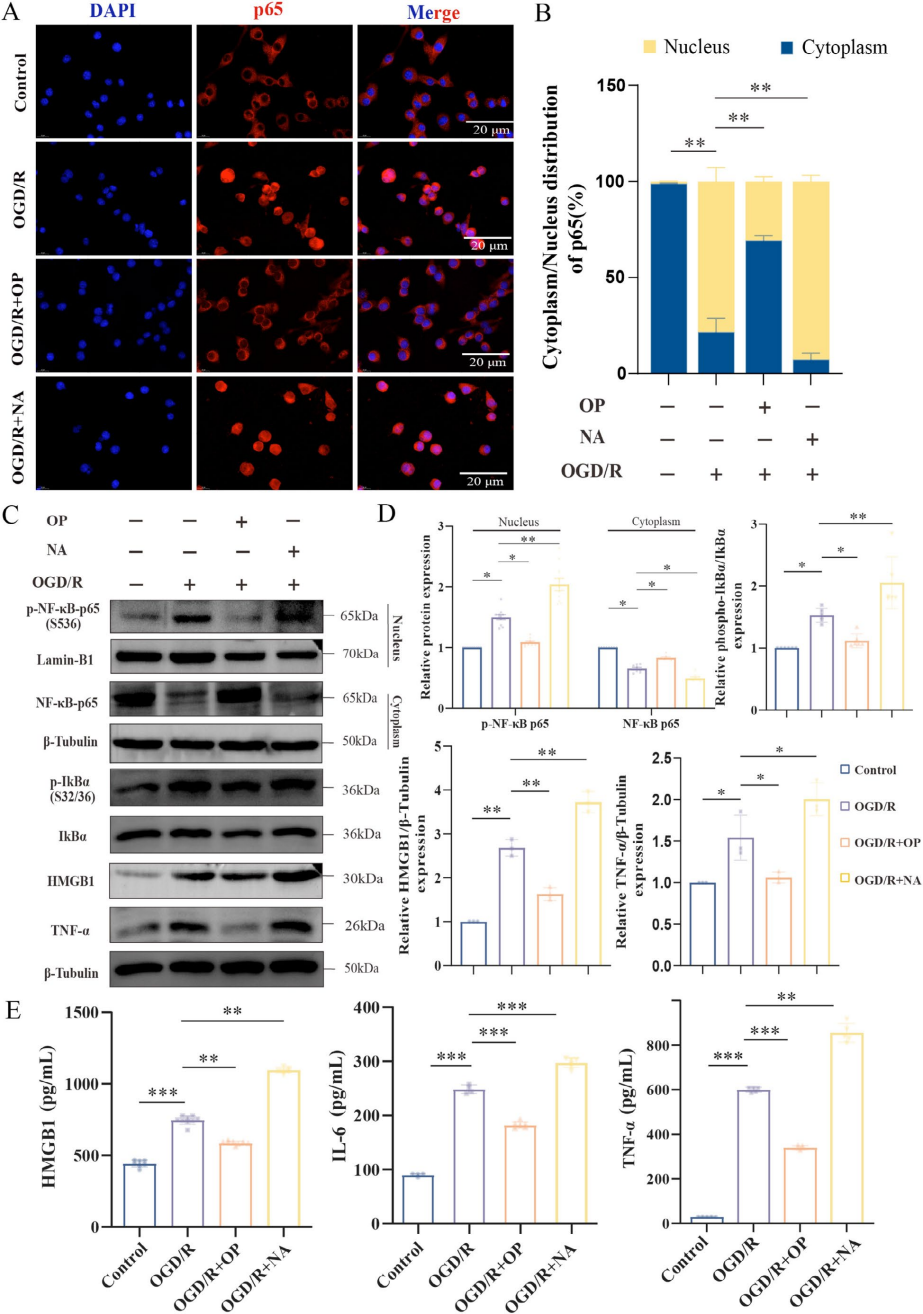
OP inhibits in vitro neuraminidase‐mediated NF‐κB pathway activation. (A) Immunofluorescence images showing NF‐κB p65 subcellular localization in BV2 microglia. Nuclei are stained with DAPI (blue), p65 is labeled with an anti‐p65 antibody (red), and merged images highlight nuclear translocation. Scale bar: 20 μm. (B) Quantification of cytoplasmic/nuclear p65 ratio in control, OGD/R, OGD/*R* + OP (40 μM), and OGD/*R* + NA (12.5 U/mL). Data expressed as a percentage of nuclear p65 distribution. (C, D) Representative Western blot (C) and quantitative analysis (D) of total NF‐κB p65, p‐NF‐κB p65 (Ser536), and Lamin‐B1, with β‐Tublin used as internal controls for nuclear and cytosolic fractions, respectively. Quantifications of p‐IκBα (Ser32/36), TNF‐α, and HMGB1 protein expression, normalized to total IκBα, and β‐Tubulin. (E) ELISA quantification of secreted HMGB1, IL‐6, and TNF‐α in BV2 microglial supernatants. NA, Neuraminidase. Values represent mean ± SEM. *p < 0.05, **p < 0.01, ***p < 0.001 (one‐way ANOVA with Bonferroni post hoc test).

To investigate the OP effects on the NF‐κB pathway in BV2 microglia under OGD/R conditions, we first assessed p65 nuclear translocation using immunofluorescence staining (Figure 5A,B). Quantitative analysis revealed that exogenous neuraminidase administration during OGD/R significantly enhanced p65 nuclear translocation, increasing the nucleocytoplasmic ratio by 14.22% ± 4.6% compared to OGD/R controls (*p* < 0.01). In contrast, OP effectively suppressed neuraminidase‐induced nuclear translocation, reducing the ratio by 47.66% ± 5.60% compared to OGD/R (*p* < 0.01).

To further validate the regulatory effects of OP on the NF‐κB pathway, we performed nuclear and cytoplasmic fractionation and analyzed p‐NF‐κB p65 (nuclear), NF‐κB p65 (cytoplasm), p‐IκBα, HMGB1, and TNF‐α levels via Western blot (Figure 5C,D). NF‐κB activation, quantified as the ratio of nuclear p‐NF‐κB p65 at S536 to Lamin‐B1, was reduced to 1.38 ± 0.10‐fold of OGD/R levels after OP treatment (*p* < 0.05). In contrast, neuraminidase treatment enhanced OGD/R‐induced p65 nuclear translocation (*p* < 0.05) and decreased cytoplasmic NF‐κB p65 levels (*p* < 0.01). OP concurrently inhibited p‐IκBα levels by 1.37 ± 0.10‐fold and reduced HMGB1 (1.65 ± 0.02‐fold) and TNF‐α (1.46 ± 0.30‐fold) secretion compared to OGD/R groups (all *p* < 0.05). Notably, neuraminidase supplementation exacerbated inflammatory signaling in OGD/R models, significantly elevating p‐IκBα levels and increasing pro‐inflammatory cytokine expressions (all *p* < 0.05).

ELISA measurements further demonstrated that OP treatment significantly reduced the secretion of HMGB1, IL‐6, and TNF‐α (all *p* < 0.001, Figure 5E). These findings confirmed that neuraminidase amplified neuroinflammation by enhancing p65 nuclear translocation and promoting cytokines release. Conversely, OP counteracted this response by blocking NF‐κB activation and suppressing HMGB1/TNF‐α production, thereby mitigating microglial hyperactivation in OGD/R models.

### 3.6 OP Preserved CD24‐Siglec‐G Interaction by Counteracting Neuraminidase‐Induced Disruption

**FIGURE 6:**
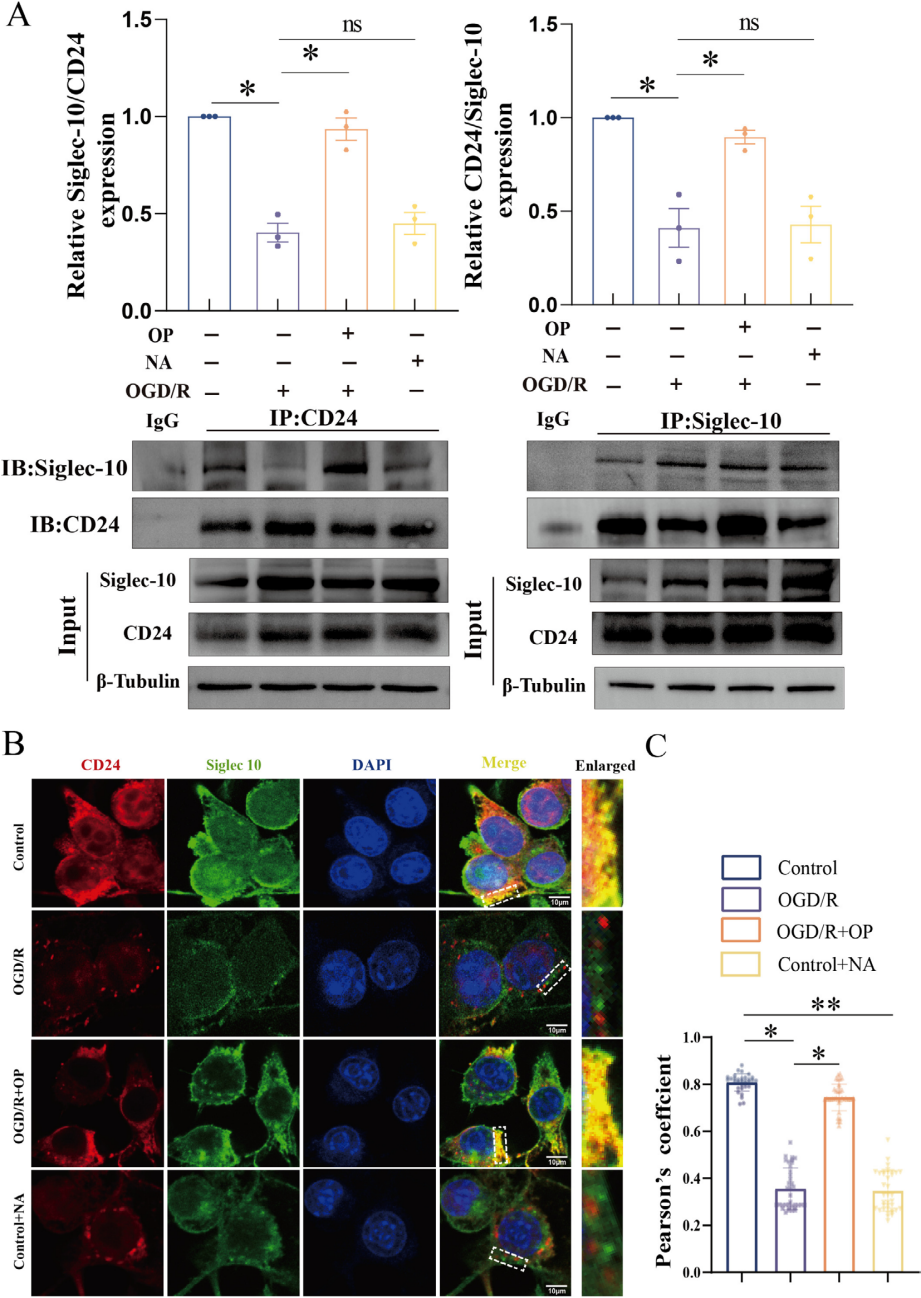
OP preserves CD24‐Siglec‐G/10 interaction by counteracting neuraminidase‐mediated desialylation. (A) Co‐immunoprecipitation (Co‐IP) and Western blot analysis of CD24 and Siglec G interactions in BV2 microglia in control, OGD/R, OGD/*R* + OP (40 μM), and Control + NA (12.5 U/mL). Lysates were immunoprecipitated with anti‐CD24 antibody and probed for Siglec‐G. Quantification of Siglec‐G and CD24 binding ratios normalized to untreated controls. (B) Dual immunofluorescence staining of CD24 (red) and Siglec‐G (green) localization in BV2 microglia. Nuclei are counterstained with DAPI (blue). Merged images demonstrate CD24‐Siglec‐G co‐localization (yellow). Scale bar: 10 μm. (C) Quantification of CD24‐Siglec‐G co‐localization using Pearson's correlation coefficient (*r*). NA, Neuraminidase. Data represent mean ± SEM. *p < 0.05, **p < 0.01 (one‐way ANOVA with Bonferroni post hoc test).

To elucidate the mechanism by which OP modulated the microglial inflammation, we investigated the interaction between CD24 and Siglec‐G using Co‐IP together with Western blot (Figure 6A). Prior SNA‐FITC lectin staining confirmed that OP effectively inhibited neuraminidase‐mediated sialic acid hydrolysis on BV2 cells. Co‐IP assays revealed robust endogenous CD24‐Siglec‐G binding in the control group, consistent with prior studies [12, 29]. However, OGD/R significantly reduced this interaction by 56.72% ± 0.20% compared to controls (all *p* < 0.05). Exogenous neuraminidase (12.5 U/mL, 37°C, 2 h) mimicked ischemic effects, decreasing CD24‐Siglec‐G binding by 47.26% ± 0.28%, a trend analogous to OGD/R‐challenged cells. Notably, OP treatment (40 μM, 48 h) restored CD24‐Siglec‐G binding to 90.20% ± 0.08% of control levels, significantly higher than in OGD/R‐challenged cells (all *p* < 0.05), indicating OP ability to stabilize immune checkpoint signaling by preventing neuraminidase‐induced de‐sialylation.

For further validation, dual immunofluorescence staining demonstrated the colocalization of CD24 (red) and Siglec‐G (green) in BV2microglia (Figure 6B,C). Quantitative colocalization analysis (Pearson's *r* = 0.81 ± 0.09 in controls) showed a significant reduction in CD24‐Siglec‐G interaction under OGD/R conditions (*r* = 0.36 ± 0.20). OP treatment restored colocalization to 92.18% ± 0.20% of control levels (*r* = 0.74 ± 0.12), underscoring the CD24‐Siglec‐G axis as a pivotal mediator of OP's anti‐inflammatory efficacy.

### 3.7 CD24 and Siglec‐G Overexpression Attenuated Neuroinflammation

**FIGURE 7:**
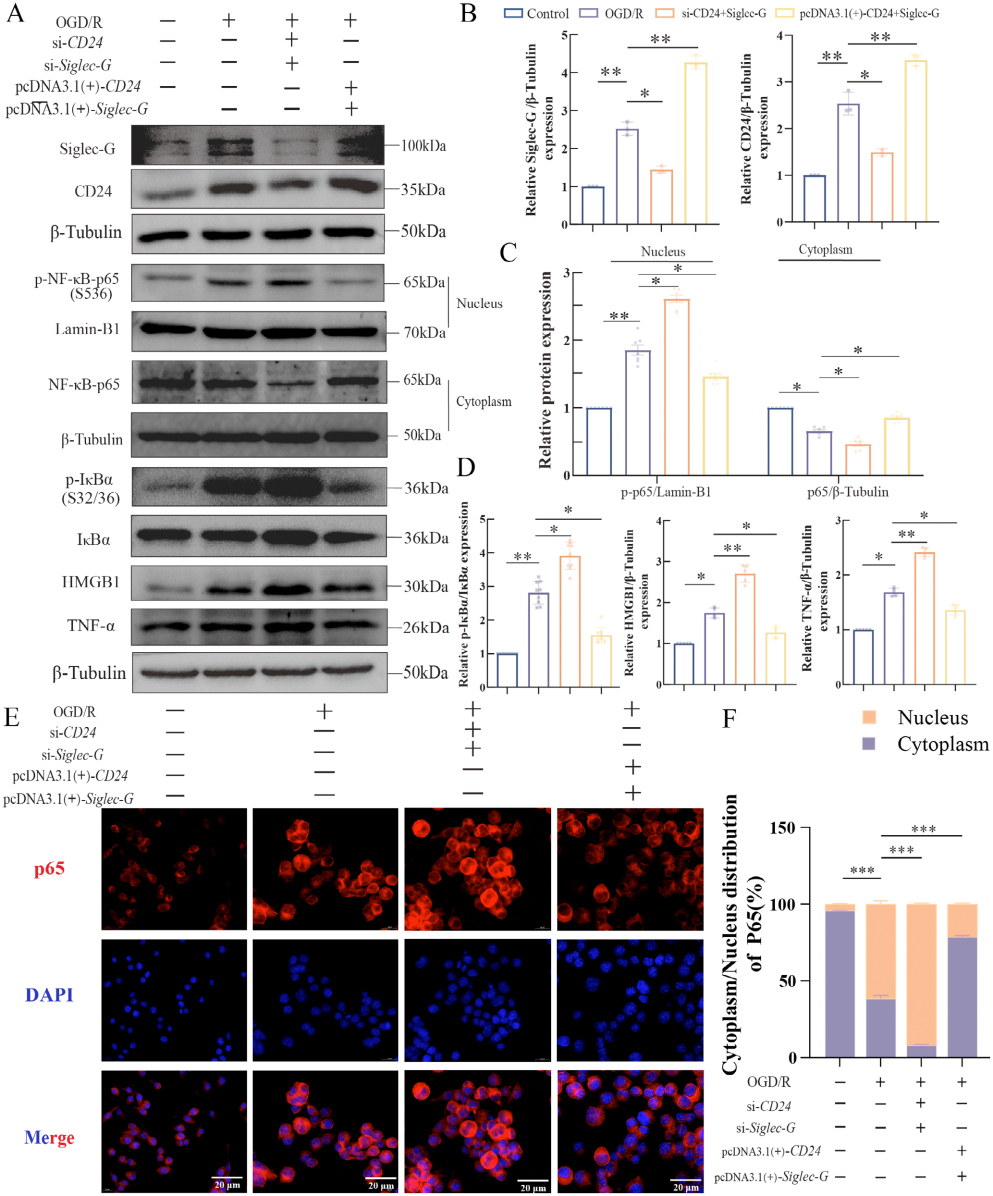
CD24 and Siglec‐G overexpression attenuates neuroinflammatory signaling in OGD/R‐injured BV2 microglia. (A) Representative western blotting bands of Siglec‐G, CD24, p‐NF‐κB‐p65 (Ser536), NF‐κB p65, p‐IκBα (Ser32/36), IκBα, TNF‐α, and HMGB protein levels in BV2 cells subjected to CD24/Siglec‐G silencing (siRNA) or overexpression (pcDNA3.1(+)) followed by OGD/R. (B) Quantitative analysis of Siglec‐G and CD24 protein levels normalized to β‐tubulin. (C) Cytoplasmic and nuclear p65 phosphorylation levels quantified as the ratio of phosphorylated to total p65. Nuclear fractions normalized to Lamin‐B1; cytoplasmic fractions normalized to β‐tubulin. (D) Quantification of p‐IκBα, total IκBα, TNF‐α, and HMGB1 protein expression normalized to β‐tubulin. (E) Immunofluorescence images illustrating NF‐κB p65 subcellular localization. Nuclei are stained with DAPI (blue), p65 is labeled with an anti‐p65 antibody (red), and merged images highlight nuclear translocation. Scale bar: 20 μm. (F) Quantification of cytoplasmic/nuclear p65 ratio in control, OGD/R, OGD/R+si‐*CD24‐Siglec‐G*, and OGD/R+ pcDNA3.1(+)‐*CD24‐Siglec‐G*. Nuclear p65 distribution data presented as percentages. Data are presented as mean ± SEM. *p < 0.05, **p < 0.01, ***p < 0.001 (one‐way ANOVA with Bonferroni post hoc test).

To investigate the roles of CD24 and Siglec‐G in post‐ROSC neuroinflammation, we employed combinatorial siRNA‐mediated gene knockdown and pcDNA3.1(+)‐based overexpression in OGD/R‐challenged BV2 microglia. Western blot analysis revealed that OGD/R significantly upregulated both CD24 (2.54 ± 0.28‐fold vs. control; *p* < 0.01) and Siglec‐G (2.52 ± 0.18‐fold vs. control; *p* < 0.01) protein levels (Figure 7A,B), aligning with our in vivo observations. CD24 overexpression further increased protein levels by 1.37 ± 0.20‐fold relative to OGD/R (*p* < 0.01), while siRNA knockdown reduced its expression to 1.70 ± 0.29‐fold of OGD/R levels (*p* < 0.05, Figure 7B). Similarly, Siglec‐G overexpression elevated protein abundance by 1.70 ± 0.20‐fold (*p* < 0.01), whereas siRNA knockdown suppressed expression to 1.75 ± 0.24‐fold of OGD/R levels (*p* < 0.05, Figure 7B).

We next evaluated NF‐κB pathway activation (Figure 7A–C). CD24‐Siglec‐G co‐overexpression significantly attenuated OGD/R‐induced nuclear p65 accumulation to 1.15 ± 0.29‐fold of OGD/R group levels (*p* < 0.05). In contrast, dual siRNA knockdown exacerbated nuclear p‐p65 accumulation (1.18 ± 0.26‐fold increase; *p* < 0.05) and reduced cytoplasmic p65 by 1.30 ± 0.05‐fold compared to OGD/R (*p* < 0.05). Consistently, CD24‐Siglec‐G co‐overexpression suppressed p‐IκBα, TNF‐α, and HMGB1 expression (all *p* < 0.05, Figure 7A,D), while co‐knockdown enhanced these pro‐inflammatory cytokines (all *p* < 0.05), confirming CD24 and Siglec‐G as negative regulators of the neuroinflammatory pathway.

Immunofluorescence imaging results corroborated these findings. Compared to the OGD/R control group, CD24‐Siglec‐G silencing increased p65 distribution by 30.58% ± 2.86% (*p* < 0.001; Figure 7E,F), whereas overexpression significantly reduced nuclear p65 accumulation by 40.28% ± 3.67% (*p* < 0.001).

### 3.8 OP Modulates NF‐κB Pathway Activity via the CD24‐Siglec‐G Axis

**FIGURE 8:**
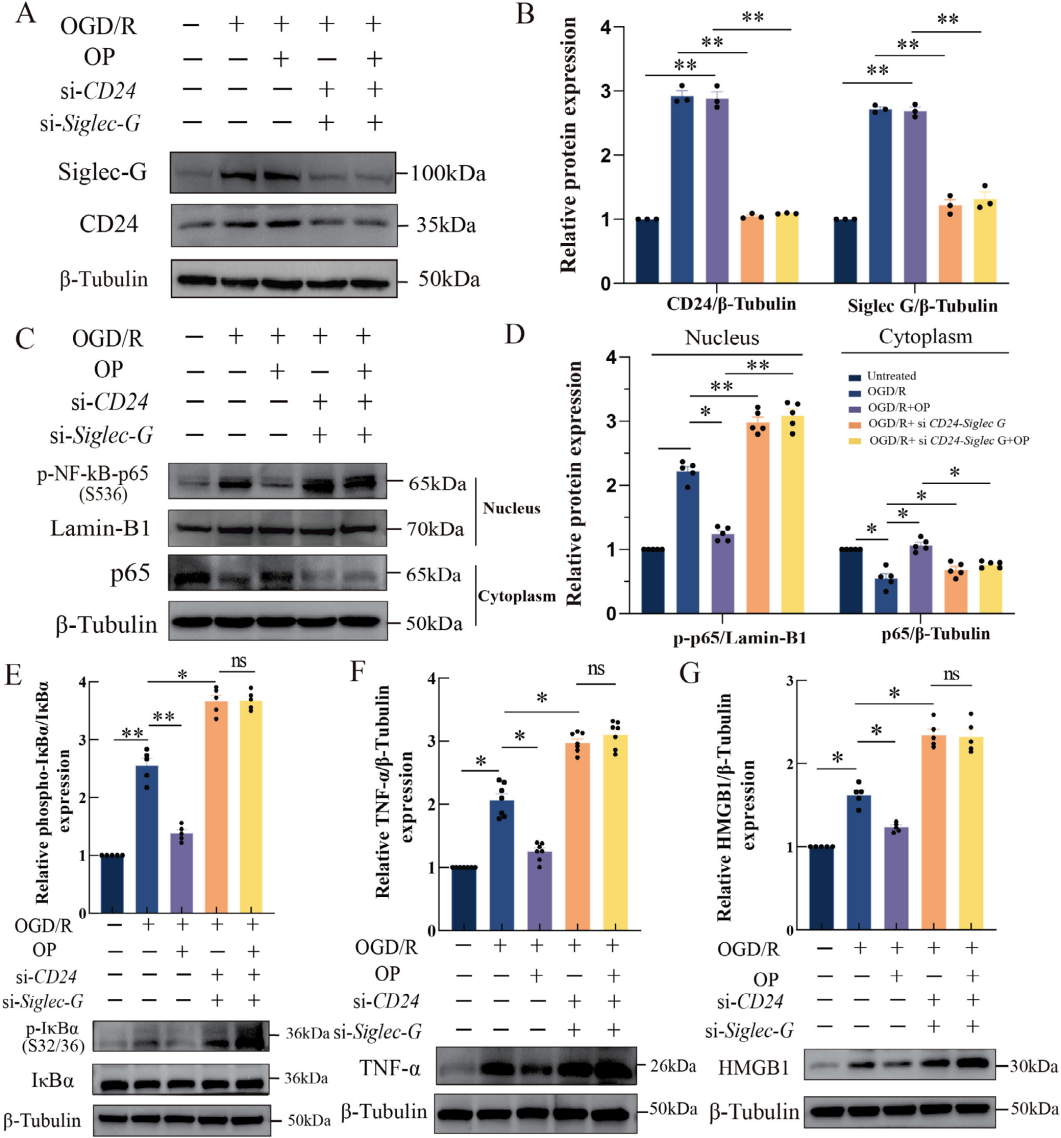
OP modulates NF‐κB pathway activity via the CD24‐Siglec‐G/10 axis in BV2 microglia. (A, B) Western blot (A) and quantitative analysis (B) of Siglec‐G and CD24 protein expressions in CD24/Siglec‐G‐silenced BV2 cells treated with OP (40 μM) following OGD/R. Protein levels normalized to β‐tubulin. (C, D) Cytoplasmic and nuclear p65 phosphorylation levels quantified as the ratio of phosphorylated to total p65. Nuclear fractions normalized to Lamin‐B1; cytoplasmic fractions normalized to β‐tubulin. (E–G) Western blot and relative quantification of p‐IκBα (Ser32/36), TNF‐α, and HMGB1 proteins, normalized to total IκBα and β‐tubulin. Values represent mean ± SEM, *p < 0.05, **p < 0.01 (two‐way ANOVA with Bonferroni post hoc test).

To further validate the CD24‐Siglec‐G axis as the mechanistic basis for OP's anti‐inflammatory effects, we performed dual knockdown of CD24 and Siglec‐G in OP‐treated BV2 microglia subjected to OGD/R (Figure 8A,B). Immunoblotting analysis revealed that CD24/Siglec‐G co‐knockdown in OGD/R‐stimulated microglia exacerbated NF‐κB activation, with nuclear p‐p65 accumulation increasing by 1.34 ± 0.08‐fold (*p* < 0.01) and cytoplasmic p65 levels decreasing by 1.60 ± 0.62‐fold (*p* < 0.05) compared to OGD/R alone (Figure 8C,D). Strikingly, OP treatment failed to rescue these effects in co‐knockdown models, showing no significant modulation of nuclear p‐p65 or cytoplasmic p65 expression (Figure 8C,D).

Co‐knockdown cells exhibited elevated p‐IκBα (1.38 ± 0.20‐fold; *p* < 0.05), HMGB1 (1.42 ± 0.46‐fold; *p* < 0.05), and TNF‐α (1.45 ± 0.28‐fold; *p* < 0.05) compared to OGD/R controls (Figure 8E–G). OP treatment normalized these inflammatory markers to baseline levels in wild‐type cells but showed no impact in co‐knockdown cells, with cytokine levels matching untreated co‐knockdown controls. The loss of OP's anti‐inflammatory activity in the absence of CD24/Siglec‐G conclusively established the CD24‐Siglec‐G axis as the critical pathway mediating OP's protective effects against ischemic injury.

## 4 Discussion

Neuroinflammation, predominantly driven by microglia hyperactivation, is a key contributor to both acute neuronal damage and chronic cognitive deficits after cerebral I/R injury [16, 37]. In the present study, we demonstrated that OP significantly attenuated neuroinflammatory responses and alleviated neuronal damage in post‐ROSC pig brain tissue and OGD/R‐challenged BV2 microglia. Mechanistically, OP exerted its neuroprotective effects by inhibiting neuraminidase activity, thereby preserving the sialylation status of CD24 and reinforcing its interaction with Siglec‐G/10.

As resident macrophages in the central nervous system, microglia maintain neuronal homeostasis but paradoxically contribute to neuropathology when dysregulated under conditions such as cerebral I/R injury [38, 39]. While their activation is essential for initiating tissue repair [40], excessive microglial responses following I/R injury often result in unintended neuronal damage through the massive inflow of pro‐inflammatory mediators [41]. In this study, we observed increased expression of p‐NF‐κB, total NF‐κB, IκBα, HMGB1, and TNF‐α in post‐ROSC pig brain tissue and OGD/R‐challenged BV2 microglia, underscoring the central role of microglial‐driven neuroinflammation. Notably, HMGB1 as a DAMP molecule could amplify inflammation by binding to TLR4 [9], thereby triggering NF‐κB nuclear translocation and perpetuating a self‐reinforcing production cycle of pro‐inflammatory cytokines [42].

Intriguingly, emerging evidence identified the CD24‐Siglec‐G/10 interaction as a regulatory checkpoint that restrains DAMP‐induced hyperinflammation [11]. By forming a tripartite complex with HMGB1 and heat‐shock protein 70/90, CD24‐Siglec‐G/10 could suppress HMGB1/TLR4/NF‐κB signaling, effectively serving as a molecular brake on immune overactivation [23, 43, 44]. Our findings aligned with this paradigm that upregulated CD24 and Siglec‐G/10 expression in post‐ROSC brain tissue and OGD/R‐challenged microglia correlated with attenuated inflammatory responses. Functional validation further revealed that CD24/Siglec‐G overexpression significantly dampened the neuroinflammatory response, whereas genetic silencing exacerbated cytokine release and NF‐κB activation.

Sialic acid serves as a critical structural component to stabilize glycoproteins on the cellular surface [45], functioning as a molecular “bridge” to facilitate the interaction between CD24 and Siglec‐G/10 [46]. As a sialylated glycoprotein, CD24 relies on its terminal sialic acid residues to engage Siglec‐G/10, thereby enforcing an immunosuppressive checkpoint [47, 48, 49]. However, neuraminidase‐mediated desialylation can disrupt this interaction, thus ablating the anti‐inflammatory capacity of CD24 and unleashing pro‐inflammatory cascades. In alignment with this mechanism, we demonstrated that neuraminidase activity was significantly upregulated in the post‐ROSC pig brains and OGD/R‐challenged BV2 microglia. Exogenous neuraminidase supplementation further exacerbated neuroinflammation, as evidenced by elevated expressions of p‐NF‐κB, total NF‐κB, IκBα phosphorylation, HMGB1 and TNF‐α. These findings directly implicated the role of neuraminidase in dismantling the sialic acid‐dependent CD24‐Siglec‐G/10 interaction, thereby derepressing NF‐κB‐driven inflammatory pathways. This phenomenon mirrored those observed in sepsis models, where neuraminidase‐induced desialylation of CD24 disrupted its binding to Siglec‐G/10, leading to unchecked NF‐κB activation and systemic hyperinflammation [12]. Our work extended this paradigm to cerebral I/R injury, identifying neuraminidase as a shared mediator of immune dysregulation across diverse inflammatory contexts [50, 51]. By cleaving sialic acid residues, neuraminidase not only compromised CD24‐Siglec‐G/10 binding but also liberated HMGB1 from its inhibitory complex, perpetuating a feedforward cycle of microglial activation and neuronal injury.

Notably, our study provided the first evidence that OP, a clinically approved neuraminidase inhibitor, could confer neuroprotection in post‐resuscitation phase by attenuating neuroinflammation and neuronal damage. In both pig CA models and OGD/R‐challenged BV2 microglia, OP administration significantly suppressed neuraminidase activity while reducing inflammatory cytokines release and microglial hyperactivation. Crucially, genetic silencing of CD24 and Siglec‐G abolished OP's ability to mitigate NF‐κB activation and inflammatory cytokines production, underscoring the necessity of the CD24‐Siglec‐G/10 axis for OP‐mediated neuroprotection. This dependency likely stems from OP's capacity to preserve CD24 sialylation by inhibiting neuraminidase, thereby stabilizing the CD24‐Siglec‐G/10 complex and its immunosuppressive function against the NF‐κB‐driven microglial hyperactivation and pro‐inflammatory cytokines release.

Intriguingly, OP exhibited dose‐ and time‐dependent neuroprotective effects. While high‐dose OP (80–100 μM) rapidly enhanced microglial viability within 24 h post‐OGD/R, lower doses (40 μM) achieved comparable efficacy by 48 h, suggesting a therapeutic window for dose optimization. The time‐dependent neuraminidase surge observed post‐ROSC in the current study (Figure 3A) could define a practical therapeutic window (< 24 h post ROSC) for OP administration, aligning with current post‐resuscitation care timelines. These findings not only elucidated a novel mechanism by which OP could modulate postischemic neuroinflammation but also positioned it as a promising candidate for drug repurposing. Traditionally utilized for influenza treatment, OP's ability to target the CD24‐Siglec‐G/10 axis offers a strategic avenue to mitigate secondary brain injury after CA, bridging the gap between virology and neurocritical care. However, a potential risk of neuropsychiatric adverse events of OP should be considered despite established safety profiles with minimal gastrointestinal disturbances as a Food and Drug Administration‐approved neuraminidase inhibitor. This was mainly from a nationwide population‐based cohort study that revealed an increased risk of neuropsychiatric adverse events after OP treatment in children and adolescents aged greater than 10 years [52].

Our study had several limitations. First, the pig CA/CPR model utilized juvenile pigs (4–6 months old), which may not fully recapitulatethe pathophysiological complexity of CA in adult or elderly populations with multiple preexisting comorbidities [53]. Our pig CA model was electrically induced ventricular fibrillation. In the clinic, patients could have CA from various causes other than ventricular fibrillation [54]. The neuroinflammation following ROSC may have distinct pathophysiological changes. These age‐ and disease‐related differences in neuroinflammatory responses and drug metabolism could influence the translational relevance of our findings. Second, while the OP dosage of 5 mg/kg was supported by preclinical evidence in severe inflammatory models [55], it requires further pharmacokinetic validation in the CA/CPR‐specific contexts to optimize dosing regimens. Third, our observations were confined to a 24‐h post‐ROSC window. Further studies should evaluate prolonged treatment regimens and dose–response relationships in human cohorts to optimize therapeutic protocols for post‐CA long‐term brain injury. Fourth, the translational efficacy of OP in nonviral inflammatory contexts remains uncertain. Although OP's primary mechanism involves neuraminidase inhibition, which is a pathway shared between viral and sterile inflammation, its generalizability and safety in noninfectious settings require rigorous validation. Fifth, CD24 and Siglec‐10 are expressed in diverse neural cell types, including neurons, astrocytes, and oligodendrocytes [45, 49]. Our results on microglia might be extrapolated to understand the neuroinflammatory cascades in other neuronal cells. In addition, further studies should investigate the systemic impact of the CD24‐Siglec‐G/10 axis on cell‐type‐specific contributions. Finally, while our study established that CD24‐Siglec‐G/10 interaction inhibited NF‐κB activation [26, 51], the precise downstream signaling mechanisms remained unexplored. Prior studies indicated that Siglec‐G/10 contained immunoreceptor tyrosine‐based inhibitory motifs (ITIMs) in its cytoplasmic domain. Upon ligand binding, these ITIMs can recruit phosphatases SHP‐1/SHP‐2 [24, 56], which can dephosphorylate key adaptor proteins (e.g., MyD88, TRAF6) in the TLR4/NF‐κB cascade. This dephosphorylation interrupts IKK complex activation and subsequent IκBα degradation [57, 58]. In our model (Figure 6), OP‐enhanced CD24‐Siglec‐G binding likely initiated this phosphatase‐driven negative feedback loop, ultimately blocking p65 nuclear translocation. Future studies should validate SHP‐1/2 recruitment and substrate dephosphorylation in microglia following OGD/R. Such investigations would elucidate the direct mechanistic link between CD24‐Siglec‐G/10 engagement and NF‐κB suppression.

## 5 Conclusion

OP, a neuraminidase inhibitor clinically approved to treat influenza, could be a promising therapeutic candidate for mitigating neuroinflammation after CA. By preserving the sialylation‐dependent interaction between CD24 and Siglec‐G/10, OP effectively suppressed NF‐κB activation in hyperactive microglia, thereby improving neuronal survival and functional recovery in postresuscitation models. Our study not only unveiled a novel mechanism underlying OP's neuroprotective properties but also highlighted its potential for repurposing in neurocritical care, diverging from a paradigm shift from its traditional antiviral applications. These insights could lay the groundwork for targeted interventions to enhance neurological outcomes in CA survivors.

## Author Contributions

Study design, experiment conduction and manuscript drafting: Yushu Chen. Data interpretation and manuscript drafting: Ying Liu and Na Li. Study conceptualization and data interpretation: Ling Wang and Peijuan Li. Statistical analysis: Zhangping Sun. ELISA assay: Dongping Yu. Manuscript revision: Ziren Tang and Ping Gong. All authors read and approved the final version of the manuscript.

## Ethics Statement

The study was conducted in strict compliance with the Guidelines for Animal Care and Use at Dalian Medical University. The Ethics Committee of Dalian Medical University approved the study (# AEE19096).

## Conflicts of Interest

The authors declare no conflicts of interest.

## Supporting information


Figure S1.

Table S1.

## Data Availability

All original data are available upon reasonable request to the corresponding author.

## References

[1] T. Henson , C. Rawanduzy , M. Salazar , et al., “Outcome and Prognostication After Cardiac Arrest,” Annals of the New York Academy of Sciences 1508 (2022): 23–34. 34580886 10.1111/nyas.14699

[2] S. S. Martin , A. W. Aday , Z. I. Almarzooq , et al., “2024 Heart Disease and Stroke Statistics: A Report of US and Global Data From the American Heart Association,” Circulation 149 (2024): e347–e913. 38264914 10.1161/CIR.0000000000001209 PMC12146881

[3] X. Xie , J. Zheng , W. Zheng , et al., “Efforts to Improve Survival Outcomes of Out‐of‐Hospital Cardiac Arrest in China: BASIC‐OHCA,” Circulation Cardiovascular Quality and Outcomes 16 (2023): e008856. 36503279 10.1161/CIRCOUTCOMES.121.008856

[4] R. L. Hoiland , P. N. Ainslie , C. L. Wellington , et al., “Brain Hypoxia Is Associated With Neuroglial Injury in Humans Post‐Cardiac Arrest,” Circulation Research 129 (2021): 583–597. 34287000 10.1161/CIRCRESAHA.121.319157 PMC8376277

[5] A. Jurcau and A. Simion , “Neuroinflammation in Cerebral Ischemia and Ischemia/Reperfusion Injuries: From Pathophysiology to Therapeutic Strategies,” International Journal of Molecular Sciences 23 (2021): 14. 35008440 10.3390/ijms23010014 PMC8744548

[6] P. Gavin , N. Robert , H. Cindy , et al., “Improving Outcomes After Post‐Cardiac Arrest Brain Injury: A Scientific Statement From the International Liaison Committee on Resuscitation,” Circulation 150 (2024). 10.1161/CIR.0000000000001219 38934122

[7] A. Castellanos‐Molina , F. Bretheau , A. Boisvert , D. Bélanger , and S. Lacroix , “Constitutive DAMPs in CNS Injury: From Preclinical Insights to Clinical Perspectives,” Brain, Behavior, and Immunity 122 (2024): 583–595. 39222725 10.1016/j.bbi.2024.07.047

[8] H. Zhang , L. Ding , T. Shen , and D. Peng , “HMGB1 Involved in Stress‐Induced Depression and Its Neuroinflammatory Priming Role: A Systematic Review,” General Psychiatry 32 (2019): e100084. 31552388 10.1136/gpsych-2019-100084 PMC6738663

[9] D. Tang , R. Kang , H. J. Zeh , and M. T. Lotze , “The Multifunctional Protein HMGB1: 50 Years of Discovery,” Nature Reviews. Immunology 23 (2023): 824–841. 10.1038/s41577-023-00894-6 37322174

[10] K. Xu , M. Wang , H. Wang , et al., “HMGB1/STAT3/p65 Axis Drives Microglial Activation and Autophagy Exert a Crucial Role in Chronic Stress‐Induced Major Depressive Disorder,” Journal of Advanced Research 59 (2024): 79–96. 37321346 10.1016/j.jare.2023.06.003 PMC11081938

[11] Y. Liu and P. Zheng , “CD24‐Siglec Interactions in Inflammatory Diseases,” Frontiers in Immunology 14 (2023): 1174789. 37228622 10.3389/fimmu.2023.1174789 PMC10203428

[12] G.‐Y. Chen , X. Chen , S. King , et al., “Amelioration of Sepsis by Inhibiting Sialidase‐Mediated Disruption of the CD24‐SiglecG Interaction,” Nature Biotechnology 29 (2011): 428–435. 10.1038/nbt.1846 PMC4090080 21478876

[13] X. Wang , M. Liu , J. Zhang , et al., “CD24‐Siglec Axis Is an Innate Immune Checkpoint Against Metaflammation and Metabolic Disorder,” Cell Metabolism 34 (2022): 1088–1103.e6. 35921817 10.1016/j.cmet.2022.07.005 PMC9393047

[14] W. Royster , P. Wang , and M. Aziz , “The Role of Siglec‐G on Immune Cells in Sepsis,” Frontiers in Immunology 12 (2021): 621627. 33708213 10.3389/fimmu.2021.621627 PMC7940683

[15] X. Fang , P. Zheng , J. Tang , and Y. Liu , “CD24: From A to Z,” Cellular & Molecular Immunology 7 (2010): 100–103. 20154703 10.1038/cmi.2009.119 PMC4001892

[16] M. Wang , W. Pan , Y. Xu , J. Zhang , J. Wan , and H. Jiang , “Microglia‐Mediated Neuroinflammation: A Potential Target for the Treatment of Cardiovascular Diseases,” Journal of Inflammation Research 15 (2022): 3083–3094. 35642214 10.2147/JIR.S350109 PMC9148574

[17] Y. Wang , H. Yu , M. Yu , et al., “CD24 Blockade as a Novel Strategy for Cancer Treatment,” International Immunopharmacology 121 (2023): 110557. 37379708 10.1016/j.intimp.2023.110557

[18] G.‐Y. Chen , N. K. Brown , P. Zheng , and Y. Liu , “Siglec‐G/10 in Self‐Nonself Discrimination of Innate and Adaptive Immunity,” Glycobiology 24 (2014): 800–806. 24996822 10.1093/glycob/cwu068 PMC4116048

[19] C. Pang , S. Gao , X.‐Z. Liu , et al., “Astrocytic CD24 Protects Neuron From Recombinant High‐Mobility Group Box 1 Protein(rHMGB1)‐Elicited Neuronal Injury,” Brain Sciences 12 (2022): 1119. 36138855 10.3390/brainsci12091119 PMC9497078

[20] T. Toubai , C. Rossi , K. Oravecz‐Wilson , et al., “Siglec‐G Represses DAMP‐Mediated Effects on T Cells,” JCI Insight 2 (2017): e92293. 28724800 10.1172/jci.insight.92293 PMC5518560

[21] T. Toubai , G. Hou , N. Mathewson , et al., “Siglec‐G‐CD24 Axis Controls the Severity of Graft‐Versus‐Host Disease in Mice,” Blood 123 (2014): 3512–3523. 24695850 10.1182/blood-2013-12-545335 PMC4041170

[22] G.‐Y. Chen , J. Tang , P. Zheng , and Y. Liu , “CD24 and Siglec‐10 Selectively Repress Tissue Damage–Induced Immune Responses,” Science 323 (2009): 1722–1725. 19264983 10.1126/science.1168988 PMC2765686

[23] Y. Liu , G.‐Y. Chen , and P. Zheng , “CD24‐Siglec G/10 Discriminates Danger‐ From Pathogen‐Associated Molecular Patterns,” Trends in Immunology 30 (2009): 557–561. 19786366 10.1016/j.it.2009.09.006 PMC2788100

[24] G.‐Y. Chen , N. K. Brown , W. Wu , et al., “Broad and Direct Interaction Between TLR and Siglec Families of Pattern Recognition Receptors and Its Regulation by Neu1,” eLife 3 (2014): e04066. 25187624 10.7554/eLife.04066 PMC4168287

[25] M. Parlato , F. Souza‐Fonseca‐Guimaraes , F. Philippart , B. Misset , M. Adib‐Conquy , and J. M. Cavaillon , “CD24‐Mediated Neutrophil Death in Inflammation: Ex Vivo Study Suggesting a Potential Role in Sepsis,” Critical Care 16 (2012): P81.

[26] J. Wißfeld , T. Abou Assale , G. Cuevas‐Rios , H. Liao , and H. Neumann , “Therapeutic Potential to Target Sialylation and SIGLECs in Neurodegenerative and Psychiatric Diseases,” Frontiers in Neurology 15 (2024): 1330874. 38529039 10.3389/fneur.2024.1330874 PMC10961342

[27] E. J. Sailor‐Longsworth , R. D. Lutze , M. A. Ingersoll , et al., “Oseltamivir (Tamiflu), a Commonly Prescribed Antiviral Drug, Mitigates Hearing Loss in Mice,” Clinical and Translational Medicine 14 (2024): e1803. 39133201 10.1002/ctm2.1803 PMC11318337

[28] T. Jefferson , M. Jones , P. Doshi , E. A. Spencer , I. Onakpoya , and C. J. Heneghan , “Oseltamivir for Influenza in Adults and Children: Systematic Review of Clinical Study Reports and Summary of Regulatory Comments,” BMJ 348 (2014): g2545. 24811411 10.1136/bmj.g2545 PMC3981975

[29] V. Y. Glanz , V. A. Myasoedova , A. V. Grechko , and A. N. Orekhov , “Inhibition of Sialidase Activity as a Therapeutic Approach,” Drug Design, Development and Therapy 12 (2018): 3431–3437. 30349196 10.2147/DDDT.S176220 PMC6186905

[30] D. Wang , L. Wang , Y. Sun , et al., “Effects of Temperature Control on Hyperthermia‐Related Cardiac Dysfunction in a Porcine Model of Cardiac Arrest,” Cryobiology 110 (2023): 49–55. 36509162 10.1016/j.cryobiol.2022.12.017

[31] P. Gong , g. Zhao , R. Hua , et al., “Mild Hypothermia Inhibits Systemic and Cerebral Complement Activation in a Swine Model of Cardiac Arrest,” Journal of Cerebral Blood Flow and Metabolism 35 (2015): 1289–1295. 25757755 10.1038/jcbfm.2015.41 PMC4528002

[32] P. Gong , R. Hua , Y. Zhang , et al., “Hypothermia‐Induced Neuroprotection Is Associated With Reduced Mitochondrial Membrane Permeability in a Swine Model of Cardiac Arrest,” Journal of Cerebral Blood Flow and Metabolism 33 (2013): 928–934. 23486294 10.1038/jcbfm.2013.33 PMC3677114

[33] L. Wang , Y. Sun , F. Kong , et al., “Mild Hypothermia Alleviates Complement C5a‐Induced Neuronal Autophagy During Brain Ischemia–Reperfusion Injury After Cardiac Arrest,” Cellular and Molecular Neurobiology 43 (2023): 1957–1974. 36006573 10.1007/s10571-022-01275-8 PMC11412180

[34] K. J. Livak and T. D. Schmittgen , “Analysis of Relative Gene Expression Data Using Real‐Time Quantitative PCR and the 2(‐Delta Delta C(T)) Method,” Methods 25 (2001): 402–408. 11846609 10.1006/meth.2001.1262

[35] B. Linnartz , J. Kopatz , A. J. Tenner , and H. Neumann , “Sialic Acid on the Neuronal Glycocalyx Prevents Complement C1 Binding and Complement Receptor‐3‐Mediated Removal by Microglia,” Journal of Neuroscience 32 (2012): 946–952. 22262892 10.1523/JNEUROSCI.3830-11.2012 PMC4037907

[36] J.‐L. Wang , X.‐Y. Hu , C.‐G. Han , S. Y. Hou , H. S. Wang , and F. Zheng , “Lanthanide Complexes for Tumor Diagnosis and Therapy by Targeting Sialic Acid,” ACS Nano 16 (2022): 14827–14837. 35981089 10.1021/acsnano.2c05715

[37] Q. Xia , G. Zhan , M. Mao , Y. Zhao , and X. Li , “TRIM45 Causes Neuronal Damage by Aggravating Microglia‐Mediated Neuroinflammation Upon Cerebral Ischemia and Reperfusion Injury,” Experimental & Molecular Medicine 54 (2022): 180–193. 35217833 10.1038/s12276-022-00734-y PMC8894463

[38] B. Linnartz , Y. Wang , and H. Neumann , “Microglial Immunoreceptor Tyrosine‐Based Activation and Inhibition Motif Signaling in Neuroinflammation,” International Journal of Alzheimer's Disease 2010 (2010): 1–7. 10.4061/2010/587463 PMC2915791 20721346

[39] G. C. Brown and A. Vilalta , “How Microglia Kill Neurons,” Brain Research 1628 (2015): 288–297. 26341532 10.1016/j.brainres.2015.08.031

[40] E. C. Wright‐Jin and D. H. Gutmann , “Microglia as Dynamic Cellular Mediators of Brain Function,” Trends in Molecular Medicine 25 (2019): 967–979. 31597593 10.1016/j.molmed.2019.08.013 PMC6829057

[41] P. Surinkaew , P. Sawaddiruk , N. Apaijai , N. Chattipakorn , and S. C. Chattipakorn , “Role of Microglia Under Cardiac and Cerebral Ischemia/Reperfusion (I/R) Injury,” Metabolic Brain Disease 33 (2018): 1019–1030. 29656335 10.1007/s11011-018-0232-4

[42] Y. Sun , M. Hei , Z. Fang , Z. Tang , B. Wang , and N. Hu , “High‐Mobility Group Box 1 Contributes to Cerebral Cortex Injury in a Neonatal Hypoxic‐Ischemic Rat Model by Regulating the Phenotypic Polarization of Microglia,” Frontiers in Cellular Neuroscience 13 (2019): 506. 31920543 10.3389/fncel.2019.00506 PMC6917666

[43] Y. Zuo , J. Wang , F. Liao , et al., “Inhibition of Heat Shock Protein 90 by 17‐AAG Reduces Inflammation via P2X7 Receptor/NLRP3 Inflammasome Pathway and Increases Neurogenesis After Subarachnoid Hemorrhage in Mice,” Frontiers in Molecular Neuroscience 11 (2018): 401. 30459553 10.3389/fnmol.2018.00401 PMC6232389

[44] W. Li , H.‐P. Ling , W.‐C. You , et al., “Elevated Cerebral Cortical CD24 Levels in Patients and Mice With Traumatic Brain Injury: A Potential Negative Role in Nuclear Factor κb/Inflammatory Factor Pathway,” Molecular Neurobiology 49 (2014): 187–198. 23881416 10.1007/s12035-013-8509-4

[45] S. S. Siddiqui , R. Matar , M. Merheb , et al., “Siglecs in Brain Function and Neurological Disorders,” Cells 8 (2019): 1125. 31546700 10.3390/cells8101125 PMC6829431

[46] X. Hu , Y. Li , Q. Chen , et al., “Sialic Acids Promote Macrophage M1 Polarization and Atherosclerosis by Upregulating ROS and Autophagy Blockage,” International Immunopharmacology 120 (2023): 110410. 37270929 10.1016/j.intimp.2023.110410

[47] A. A. Barkal , R. E. Brewer , M. Markovic , et al., “CD24 Signalling Through Macrophage Siglec‐10 Is a Target for Cancer Immunotherapy,” Nature 572 (2019): 392–396. 31367043 10.1038/s41586-019-1456-0 PMC6697206

[48] D. C. Ayre , N. K. Pallegar , N. A. Fairbridge , M. Canuti , A. S. Lang , and S. L. Christian , “Analysis of the Structure, Evolution, and Expression of CD24, an Important Regulator of Cell Fate,” Gene 590 (2016): 324–337. 27259665 10.1016/j.gene.2016.05.038

[49] D. T. Gilliam , V. Menon , N. P. Bretz , and J. Pruszak , “The CD24 Surface Antigen in Neural Development and Disease,” Neurobiology of Disease 99 (2017): 133–144. 27993646 10.1016/j.nbd.2016.12.011

[50] M. Duan , Y. Xu , Y. Li , H. Feng , and Y. Chen , “Targeting Brain‐Peripheral Immune Responses for Secondary Brain Injury After Ischemic and Hemorrhagic Stroke,” Journal of Neuroinflammation 21 (2024): 102. 38637850 10.1186/s12974-024-03101-y PMC11025216

[51] J. C. Paulson and N. Kawasaki , “Sialidase Inhibitors DAMPen Sepsis,” Nature Biotechnology 29 (2011): 406–407. 10.1038/nbt.1859 PMC3565534 21552240

[52] H.‐R. Kang , S.‐C. Jang , and J.‐Y. Shin , “Association Between Oseltamivir Use and Neuropsychiatric Adverse Events in Influenza Patients: A Nationwide Population‐Based Cohort Study,” Expert Opinion on Drug Safety 20 (2021): 245–253. 33183123 10.1080/14740338.2021.1850690

[53] G. Hirlekar , T. Karlsson , S. Aune , et al., “Survival and Neurological Outcome in the Elderly After In‐Hospital Cardiac Arrest,” Resuscitation 118 (2017): 101–106. 28736324 10.1016/j.resuscitation.2017.07.013

[54] L. W. Andersen , M. J. Holmberg , K. M. Berg , M. W. Donnino , and A. Granfeldt , “In‐Hospital Cardiac Arrest: A Review,” JAMA 321 (2019): 1200–1210. 30912843 10.1001/jama.2019.1696 PMC6482460

[55] H. K. Ashar , S. Pulavendran , J. M. Rudd , et al., “Administration of a CXC Chemokine Receptor 2 (CXCR2) Antagonist, SCH527123, Together With Oseltamivir Suppresses NETosis and Protects Mice From Lethal Influenza and Piglets From Swine‐Influenza Infection,” American Journal of Pathology 191 (2021): 669–685. 33453177 10.1016/j.ajpath.2020.12.013 PMC8027923

[56] H. Wang , P. Shi , X. Shi , Y. Lv , H. Xie , and H. Zhao , “Surprising Magic of CD24 Beyond Cancer,” Frontiers in Immunology 14 (2024): 1334922. 38313430 10.3389/fimmu.2023.1334922 PMC10834733

[57] N. Wang , S. Tan , H. Liu , et al., “SHP‐1 Alleviates Acute Liver Injury Caused by *Escherichia coli* Sepsis Through Negatively Regulating the Canonical and Non‐Canonical NFκB Signaling Pathways,” International Immunopharmacology 143 (2024): 113371. 39413645 10.1016/j.intimp.2024.113371

[58] Q. Lv , Y. Wang , W. Tian , et al., “Exosomal miR‐146a‐5p Derived From Human Umbilical Cord Mesenchymal Stem Cells Can Alleviate Antiphospholipid Antibody‐Induced Trophoblast Injury and Placental Dysfunction by Regulating the TRAF6/NF‐κB Axis,” J Nanobiotechnology 21 (2023): 419. 37957714 10.1186/s12951-023-02179-5 PMC10641965

