# Supplementary material for: Oseltamivir Phosphate Modulates CD24‐Siglec‐G/10 Interaction to Suppress Microglial‐Driven Neuroinflammation After Cardiac Arrest
Source: CNS Neurosci Ther. 2025 Aug 21;31(8):e70495. doi: 10.1111/cns.70495 (PMC12368432; doi:10.1111/cns.70495)
Supplement: Supplementary file 1 — Figure S1. [file CNS-31-e70495-s002.zip › cns70495-sup-0002-SupplementaryFile1.docx]

**Table S1 Transfection sequences.**

| Gene | Sense（5′-3′） | | Antisense（5′-3′） |
| --- | --- | --- | --- |
| CD24-siRNA  Siglec-G-siRNA | | GCAACCACAAGUCCAAUGUTT  GUUCCUUGCUAAUCAAAGATT | ACAUUGGACUUGUGGUUGCTT  UCUUUGAUUAGCAAGGAACTT |

**Table S2 Primer sequences.**

| Gene | Sequence（5′-3′） |
| --- | --- |
| Sus scrofa TNF-α-F  Sus scrofa TNF-α-R  Sus scrofa- HMGB1-F  Sus scrofa- HMGB1-R  Sus scrofa- IL-6-F  Sus scrofa- IL-6-R  Sus scrofa-ACTB-F  Sus scrofa-ACTB-R | GGCCCAAGGACTCAGATCAT  CTGTCCCTCGGCTTTGACAT  TCTGGGTTCAATCAGGAGACC  ATCTGCACAGCCTCGACATT  TCTGGGTTCAATCAGGAGACC  ATCTGCACAGCCTCGACATT  TTCTAGGCGGACTTGCAGC  GCTTCTCAGCAGACAGGAGG |

**Table S3 Antibodies.**

| **Antibody** | **Application** | **Manufacturer** | **Catalog number** |
| --- | --- | --- | --- |
| p-IκBα,Ser32/36  IκBα  p-p65, Ser536  p65  HMGB1  Iba1  TNF-α  CD24  CD24  Siglec-10  Siglec-10  β-Tubulin  Lamin-B1 | WB  WB  WB  WB, IF  WB, IF  WB, IF  WB  WB, IP  IF  WB, IP  IF  WB  WB | CST  CST  CST  CST  Proteintech  Proteintech  Proteintech  Abcam  Abcam  CST  Biorbyt  Proteintech  Proteintech | #2859  #9242  #3033  #6956  10829-1-AP  10904-1-AP  60291-1-Ig  ab179821  ab290730  #41262  orb4863  66240-1-Ig  12987-1-AP |

**Table S4 Baseline characteristics**

|  | **Sham** | **CPR** | **CPR+OP** | ***P-value*** |
| --- | --- | --- | --- | --- |
|  | （*n*=5） | （*n*=5） | （*n*=5） |  |
| **Weight** (kg) | 30.1±1.7 | 30.0±2.0 | 30.2±1.8 | 0.873 |
| **Temperature** (bladder, ℃) | 37.2±0.4 | 37.2±0.2 | 37.2±0.3 | 0.689 |
| **Heart rate** (bpm) | 97.7±3.0 | 98±2.3 | 96.8±3.5 | 0.465 |
| **Map** (mmHg) | 105.4±3.3 | 106.8±2.9 | 106.5±2.7 | 0.663 |
| **Arterial lactate**  (mmol/L) | 1.3±0.1 | 1.4±0.2 | 1.3±0.4 | 0.412 |
| **ETCO_2_** (mmHg) | 40.5±3.4 | 40.7±3.7 | 40.2±2.5 | 0.943 |
| **P_a_O_2_** (mmHg) | 95.8±1.5 | 95.4±1.4 | 95.6±1.2 | 0.847 |

Values are mean ± SD.

CPR, cardiopulmonary resuscitation; OP, oseltamivir phosphate; bpm, beats per minute; MAP, mean aortic pressure; ETCO_2_, end-tidal carbon dioxide; P_a_O_2_, arterial oxygen partial pressure.
