# Supplementary figures and images for: Oseltamivir Phosphate Modulates CD24‐Siglec‐G/10 Interaction to Suppress Microglial‐Driven Neuroinflammation After Cardiac Arrest
Source: CNS Neurosci Ther. 2025 Aug 21;31(8):e70495. doi: 10.1111/cns.70495 (PMC12368432; doi:10.1111/cns.70495)

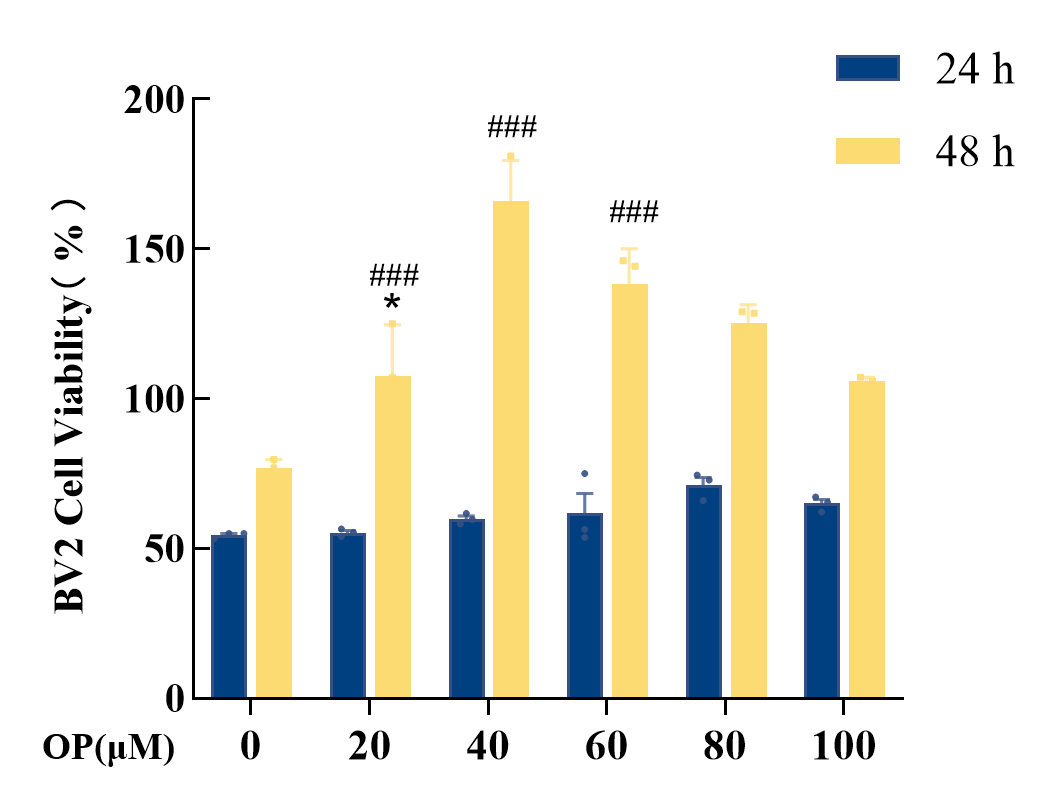

Supplement: Supplementary file 1 — Figure S1. [file CNS-31-e70495-s002.zip › cns70495-sup-0001-FigureS1.tif]
