# Supplementary material for: Oseltamivir Phosphate Modulates CD24‐Siglec‐G/10 Interaction to Suppress Microglial‐Driven Neuroinflammation After Cardiac Arrest
Source: CNS Neurosci Ther. 2025 Aug 21;31(8):e70495. doi: 10.1111/cns.70495 (PMC12368432; doi:10.1111/cns.70495)
Supplement: Supplementary file 2 — Table S1. [file CNS-31-e70495-s001.pdf]

Full-length Western blot images for Fig.2

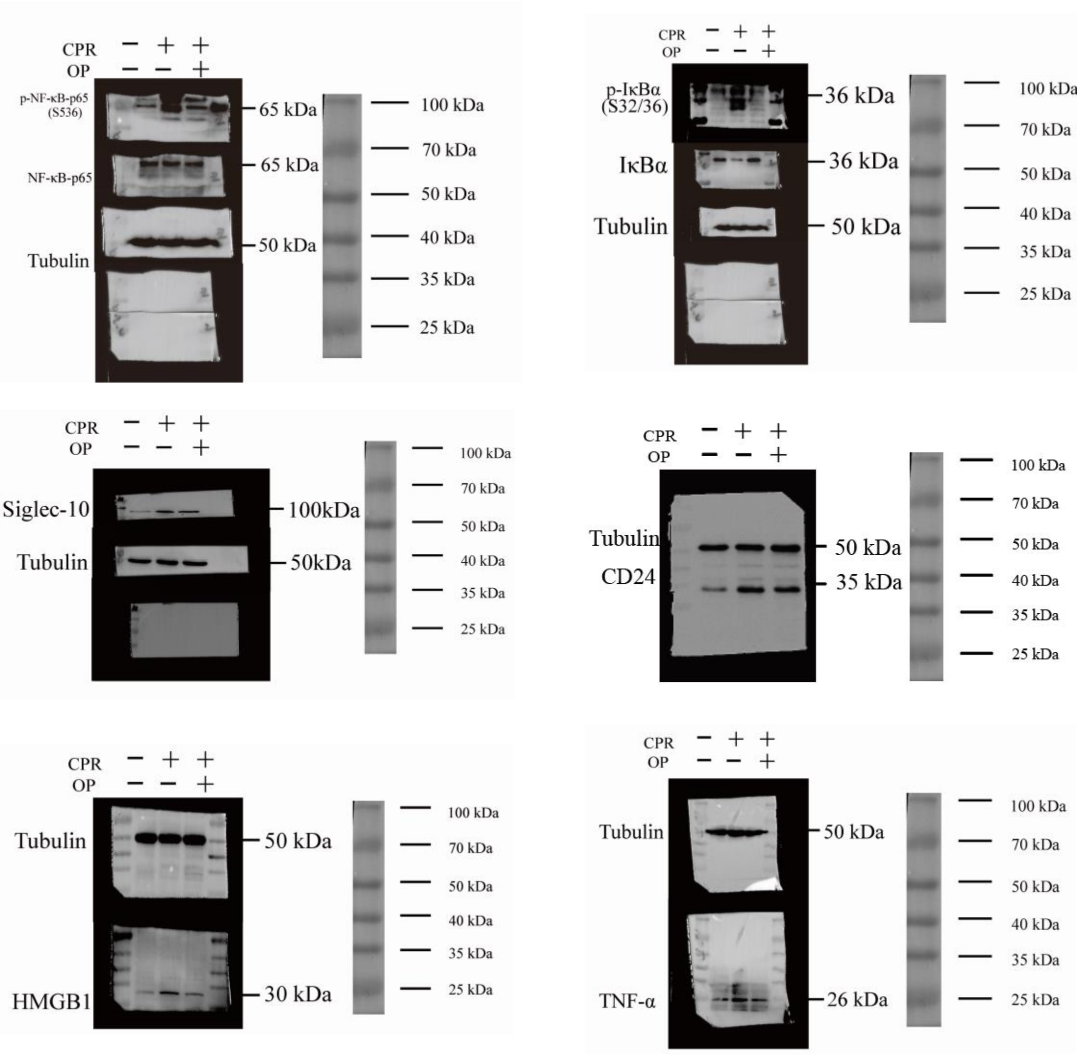

Full-length Western blot images for Fig.5

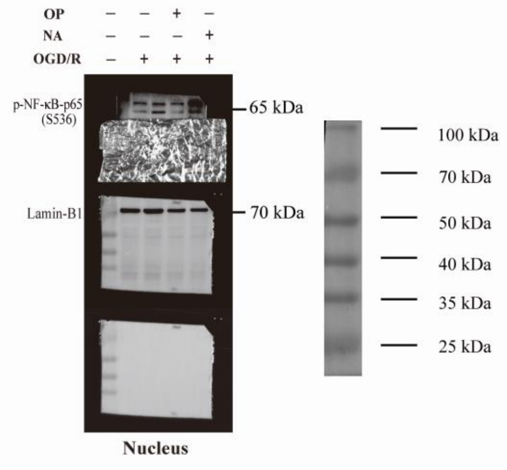

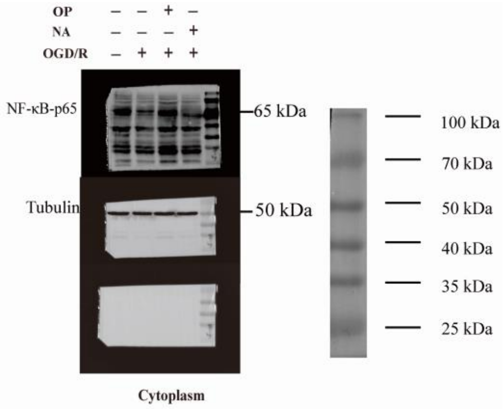

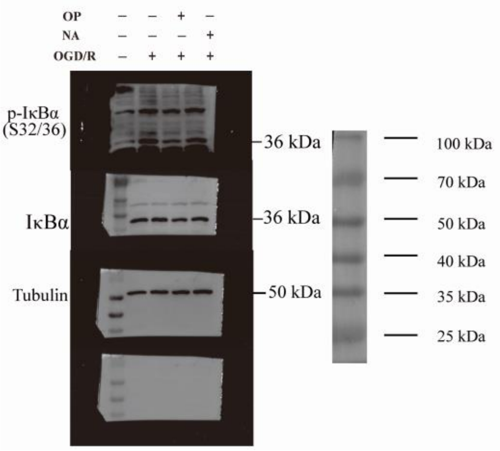

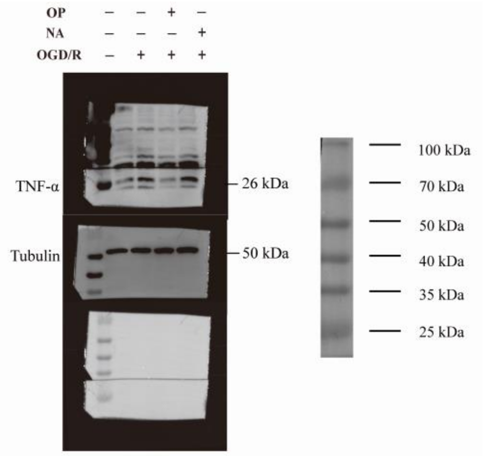

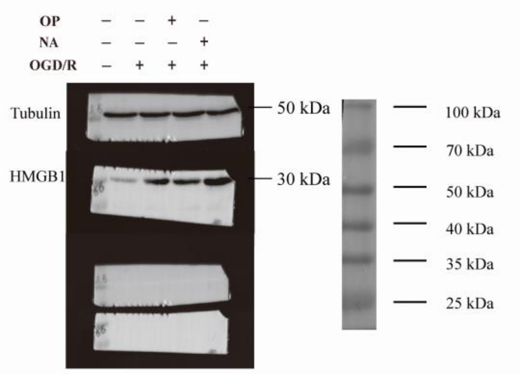

Full-length Western blot images for Fig.6

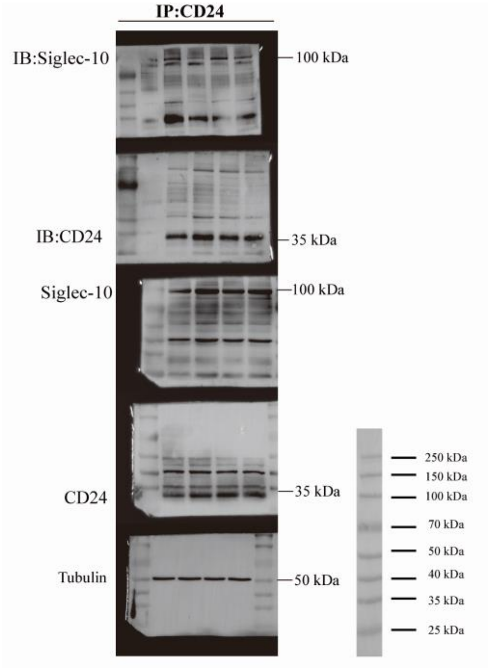

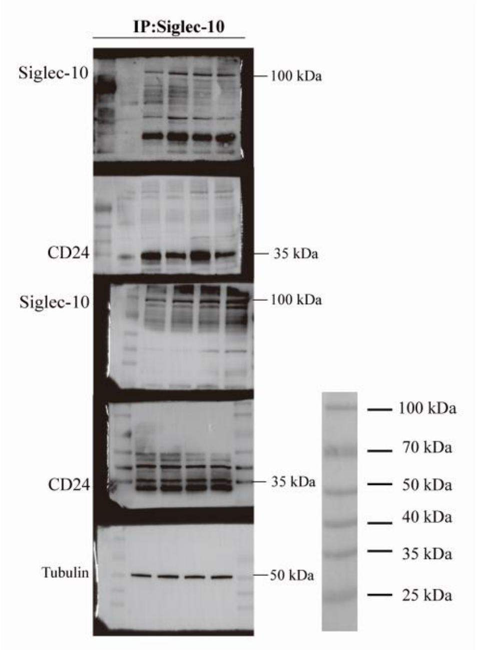

Full-length Western blot images for Fig.7

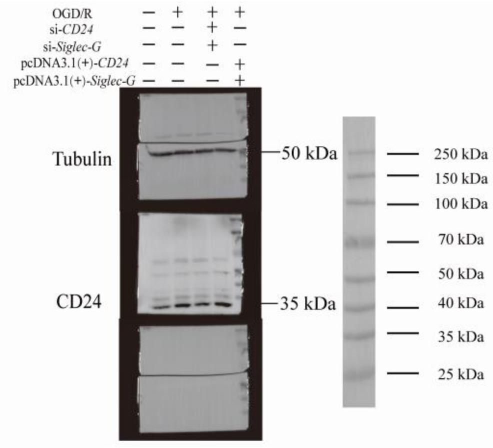

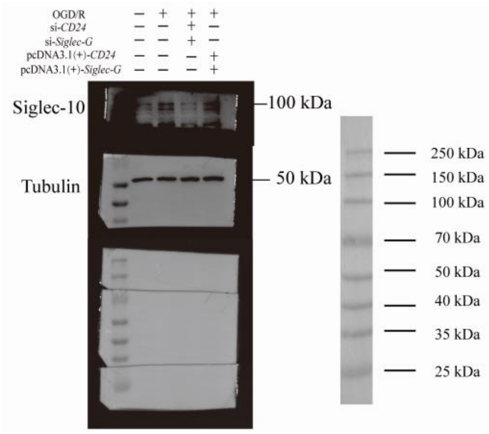

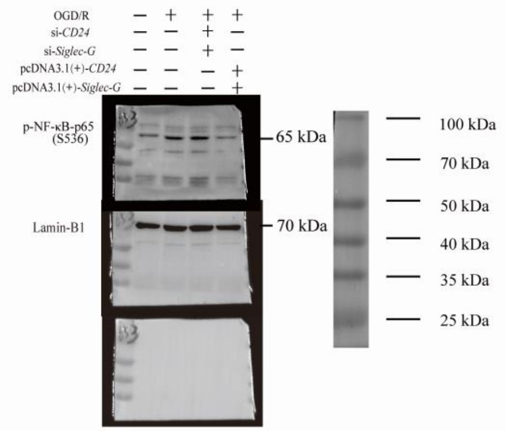

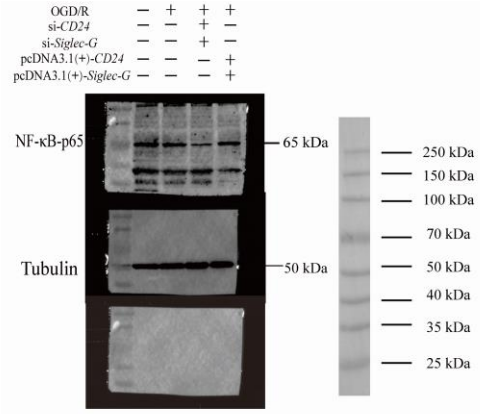

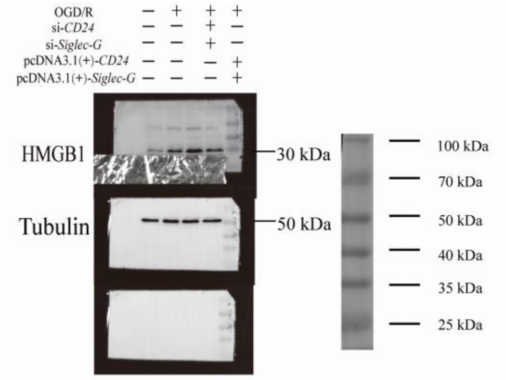

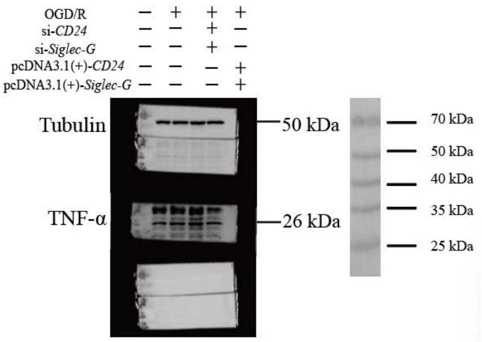

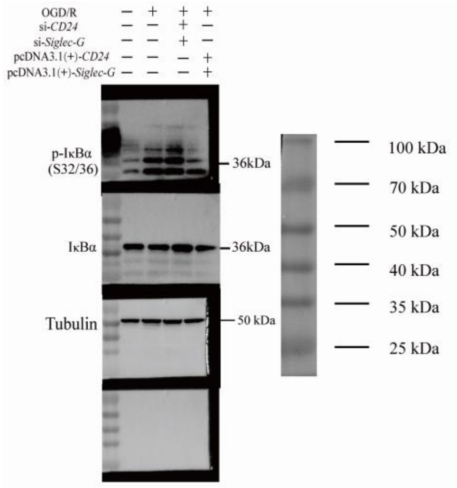

Full-length Western blot images for Fig.8

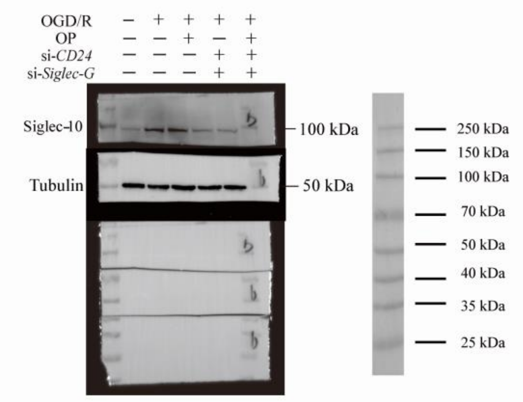

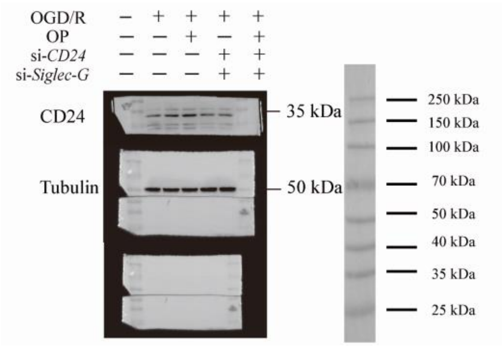

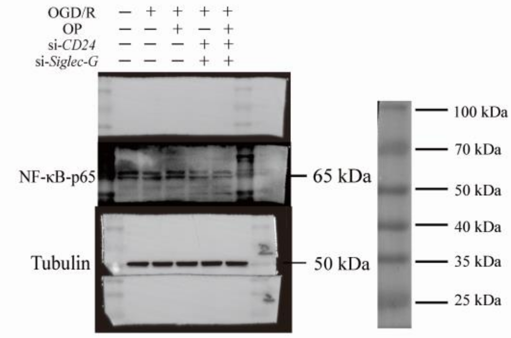

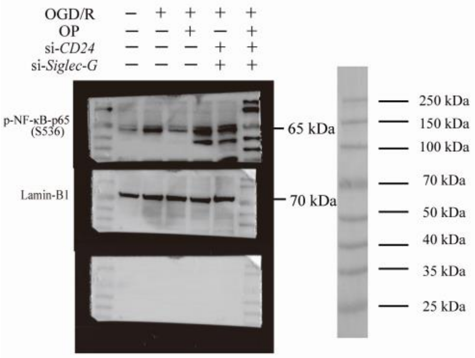

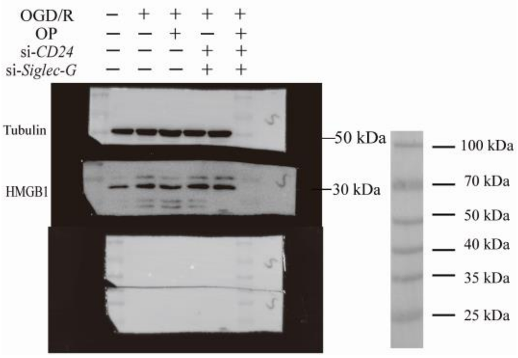
OGD/R − + + + +
OP − − + − +
si-CD24 − − − + +
si-Siglec-G − − − + +
Tubulin
50 kDa
HMGB1
30 kDa
100 kDa
70 kDa
50 kDa
40 kDa
35 kDa
25 kDa

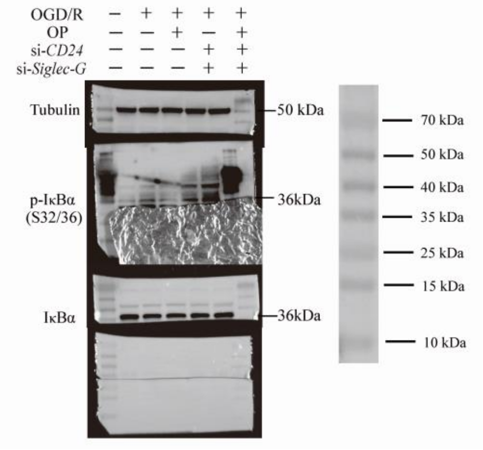
OGD/R − + + + +
OP − − + − +
si-CD24 − − − + +
si-Siglec-G − − − + +
Tubulin
50 kDa
p-IκBα (S32/36)
36kDa
IκBα
36kDa
70 kDa
50 kDa
40 kDa
35 kDa
25 kDa
15 kDa
10 kDa

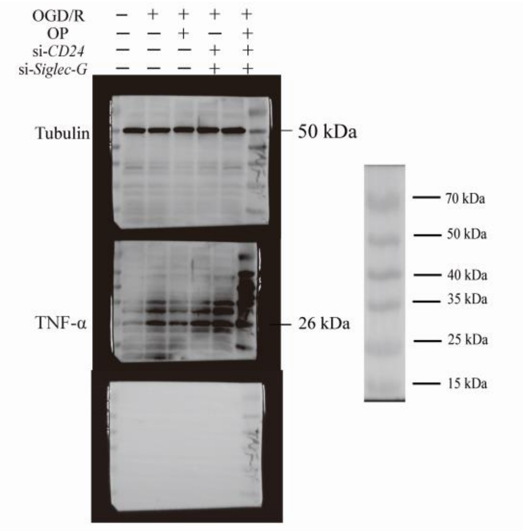
OGD/R − + + + +
OP − − + − +
si-CD24 − − − + +
si-Siglec-G − − − + +
Tubulin
50 kDa
TNF-α
26 kDa
70 kDa
50 kDa
40 kDa
35 kDa
25 kDa
15 kDa
